# Activin A and CCR2 regulate macrophage function in testicular fibrosis caused by experimental autoimmune orchitis

**DOI:** 10.1007/s00018-022-04632-4

**Published:** 2022-11-24

**Authors:** Wei Peng, Artem Kepsch, Till O. Kracht, Hiba Hasan, Rukmali Wijayarathna, Eva Wahle, Christiane Pleuger, Sudhanshu Bhushan, Stefan Günther, A. Christine Kauerhof, Ana Planinić, Daniela Fietz, Hans-Christian Schuppe, Małgorzata Wygrecka, Kate L. Loveland, Davor Ježek, Andreas Meinhardt, Mark P. Hedger, Monika Fijak

**Affiliations:** 1 Department of Anatomy and Cell Biology, Justus Liebig University of Giessen, Aulweg 123, 35392 Giessen, Germany; 2 Centre for Reproductive Health, Hudson Institute of Medical Research, Clayton, VIC Australia; 3 Department of Molecular and Translational Sciences, Monash University, Clayton, VIC Australia; 4 ECCPS Bioinformatics and Deep Sequencing Platform, Max Planck Institute for Heart and Lung Research, Bad Nauheim, Germany; 5 Department of Histology and Embryology, School of Medicine, University of Zagreb, Zagreb, Croatia; 6 Centre of Excellence for Reproductive and Regenerative Medicine, School of Medicine, University of Zagreb, Zagreb, Croatia; 7 Department of Veterinary Anatomy, Histology and Embryology, Justus Liebig University of Giessen, Giessen, Germany; 8 Department of Urology, Paediatric Urology and Andrology, Justus Liebig University of Giessen, Giessen, Germany; 9 Center for Infection and Genomics of the Lung, German Center for Lung Research, University of Giessen and Marburg Lung Center, Giessen, Germany

**Keywords:** Testicular inflammation, Fibrosis, EAO, Macrophages, CCR2, Activin A, CXCR4, MMP2

## Abstract

Experimental autoimmune-orchitis (EAO), a rodent model of chronic testicular inflammation and fibrosis, replicates pathogenic changes seen in some cases of human spermatogenic disturbances. During EAO, increased levels of pro-inflammatory and pro-fibrotic mediators such as TNF, CCL2, and activin A are accompanied by infiltration of leukocytes into the testicular parenchyma. Activin A levels correlate with EAO severity, while elevated CCL2 acting through its receptor CCR2 mediates leukocyte trafficking and recruits macrophages. CCR2 + CXCR4 + macrophages producing extracellular matrix proteins contribute widely to fibrogenesis. Furthermore, testicular macrophages (TMs) play a critical role in organ homeostasis. Therefore, we aimed to investigate the role of the activin A/CCL2-CCR2/macrophage axis in the development of testicular fibrosis. Following EAO induction, we observed lower levels of organ damage, collagen deposition, and leukocyte infiltration (including fibronectin^+^, collagen I^+^ and CXCR4^+^ TMs) in *Ccr2*^*−/−*^ mice than in *WT* mice. Furthermore, levels of *Il-10*, *Ccl2*, and the activin A subunit *Inhba* mRNAs were lower in *Ccr2*^*−/−*^ EAO testes. Notably, fibronectin^+^ TMs were also present in biopsies from patients with impaired spermatogenesis and fibrotic alterations. Overexpression of the activin A antagonist follistatin reduced tissue damage and collagen I^+^ TM accumulation in *WT* EAO testes, while treating macrophages with activin A in vitro increased the expression of *Ccr2*, *Fn1*, *Cxcr4,* and *Mmp2* and enhanced migration along a CCL2 gradient; these effects were abolished by follistatin. Taken together, our data indicate that CCR2 and activin A promote fibrosis during testicular inflammation by regulating macrophage function. Inhibition of CCR2 or activin A protects against damage progression, offering a promising avenue for therapeutic intervention.

**Supplementary Information:**

The online version contains supplementary material available at 10.1007/s00018-022-04632-4.

## Introduction

Approximately 8–12% of couples worldwide suffer from infertility. Male factors account for ~ 50% of cases, while testicular inflammation induced by bacteria, viruses, or sterile inflammation (including autoimmune diseases) is a significant cause of male infertility [1–4]. Experimental autoimmune orchitis (EAO), a rodent model of chronic testicular inflammation, mimics the pathological changes observed in human testicular biopsies from infertile patients [5–7]. In the mouse, EAO leads to fibrosis and subsequent infertility [6, 8]. Pathological features of EAO include the destruction of the testicular structure, infiltration of the interstitium by leukocytes, elevated levels of pro-inflammatory cytokines (including C–C motif chemokine ligand 2 [CCL2], tumor necrosis factor [TNF], and activin A), as well as the loss of germ cells. Furthermore, levels of C–C motif chemokine receptor type 2 (CCR2), the receptor for CCL2, are significantly increased in interstitial mononuclear cells in EAO testis [9]. Testicular macrophages (TMs) constitute the major immune cell population in the testis and play a critical role in supporting steroidogenesis, promoting spermatogenesis, and possibly also maintaining immune privilege [10, 11]. During testicular inflammation, numbers of infiltrating monocytes and macrophages are increased, while a reduction in TMs correlates with decreased incidence and severity of testicular damage [12–14].

Fibrosis is a hallmark of progressive and severe EAO, characterized by the accumulation of extracellular matrix (ECM) proteins such as collagen and fibronectin and thickening of the α-smooth muscle actin (αSMA)-positive peritubular cell (PTC) layer of the seminiferous tubules [13]. Studies in other organs have indicated that ECM-producing cells may originate not only from resident mesenchymal fibroblasts but also from circulating hematopoietic cells or cells undergoing epithelial/endothelial-to-mesenchymal transition [15]. Monocytes and macrophages that are resident or recruited to injury sites produce a variety of factors, including transforming growth factor-β (TGF-β), platelet-derived growth factors (PDGFs), and matrix metalloproteinases (MMPs). These proteins stimulate the proliferation, differentiation, and activation of fibroblasts, thereby promoting fibrotic remodeling [16, 17]. Tissue inhibitors of metalloproteinases (TIMPs) also play a role in fibrosis by inhibiting MMPs, thereby suppressing ECM proteolysis, TGF-β release, and neutrophil chemotaxis [18].

Activin A, a homodimeric member of the TGF-β superfamily of cytokines, is produced mainly by Sertoli cells (SCs) in the normal postnatal testes and plays multiple biological roles in inflammation, immunity, and fibrosis [19, 20]. In EAO, serum and testicular activin A levels are increased [13, 21]. Activin A exerts both pro- and anti-inflammatory effects and stimulates monocytes and macrophages to produce many inflammatory mediators, including interleukin (IL)-1β, IL-6, and TNF [22, 23]. Furthermore, activin A promotes pro-fibrotic gene expression in many cell types, including PTCs and NIH 3T3 fibroblasts [24]. Activin A also drives the chemotactic migration and adhesion of L929 fibroblasts in vitro [25]. Activin activity is inhibited by follistatin (FST), an endogenous high-affinity activin A binding antagonist [26].

CCL2 and CCR2 play a critical role in the trafficking of lymphocytes, monocytes, macrophages, and bone marrow-derived fibroblasts to sites of injury [27, 28]. In addition to mediating chemotaxis, CCL2 stimulates the production of collagens by fibroblasts and TGF-β by macrophages [29]. CCR2^+^ cells also contribute to fibrogenesis by producing TIMP1, which inhibits collagen degradation [30]. In the EAO model, increased levels of CCL2 protein were observed in mononuclear and endothelial cells, Leydig cells, and PTCs as well as in testicular fluid and conditioned medium from cultured TMs; furthermore, elevated expression of CCR2 was identified in mononuclear cells [9]. Of note, stimulation of Sertoli cells with TNF led to upregulated levels of *Ccl2* and activin A (*Inhba)* mRNA expression [24, 31].

At present, the role of the activin A/CCL2-CCR2/macrophage axis in this inflammatory and fibrotic response in the testis is unknown. We hypothesized that the interaction of activin A and CCR2 may influence the development of testicular fibrosis by regulating the properties of macrophages, a potentially important source of pro-fibrotic factors during orchitis. To verify our hypothesis, we compared the progress of EAO in wild type (*WT*) and *Ccr2*^*−/−*^ mutant mice, which lack peripheral blood monocytes, as well as in mice overexpressing follistatin, which display reduced activin bioactivity. Furthermore, we investigated the influence of activin A on CCR2 and fibrotic mediator expression in macrophages, using bone marrow-derived macrophages (BMDMs) matured in the presence of macrophage-colony stimulating factor (M-CSF) as a surrogate for TMs.

## Materials and methods

### Animals

In this study, 10- to 12-week-old *WT C57BL/6J* (Charles River Laboratories, Sulzfeld, Germany) and *B6.129P2-Ccr2*^*tm1Mae/tm1Mae*^ (*Ccr2*^*−/−*^) mice were housed in specific pathogen-free conditions (12 h light/dark cycle, 20–22 °C) at the animal facility of Justus Liebig University, Giessen, Germany [32]. The mice had access to water and food ad libitum. Femurs and tibias were collected from male adult *WT* and *Ccr2*^*−/−*^ mice for the isolation of bone marrow progenitor cells, while testes were obtained from 21-day-old immature *WT* mice for the isolation of SCs.

Animal experiments were approved by the responsible local ethics committees on animal care (Regierungspraesidium Giessen GI 58/2014—Nr. 735-GP and the Monash Medical Centre Animal Experimentation Committee). All experiments involving animals were carried out in strict accordance with the recommendations in the guide for the Care and Use of Laboratory Animals of the German animal welfare law and the Australian Code for the Care and Use of Animals for Scientific Purposes.

### Induction of EAO in WT and Ccr2^−/−^ mice

Peri-procedural analgesia in mice was provided by supplying tramadol (STADApharm GmbH, Bad Vilbel, Germany) in drinking water (2.5 mg/ml), commencing 24 h before each immunization and continuing for the following 3 days.

For the induction of EAO, adult male *C57BL/6 J* and *Ccr2*^*−/−*^ mice (n = 29) were first anesthetized by administration of 3–5% isoflurane and then actively immunized with testicular homogenate (TH) in complete Freund’s adjuvant (CFA; Sigma-Aldrich, St. Louis, USA), as previously described [13]. The homogenate was prepared from decapsulated testes collected from adult *WT* mice and homogenized in sterile 0.9% NaCl at a ratio of 1:1. Animals were immunized dorsally three times every 14 days with a mixture of TH in CFA; a total volume of 200 µl was delivered as four subcutaneous injections (50 µl per injection site). This was followed by intraperitoneal injection of 100 ng *Bordetella pertussis* toxin (Calbiochem, Darmstadt, Germany) in 100 µl Munõz Buffer (25 mM Tris, 0.5 M NaCl, 0.017% Triton X-100, pH 7.6) [33].

**Fig. 1:**
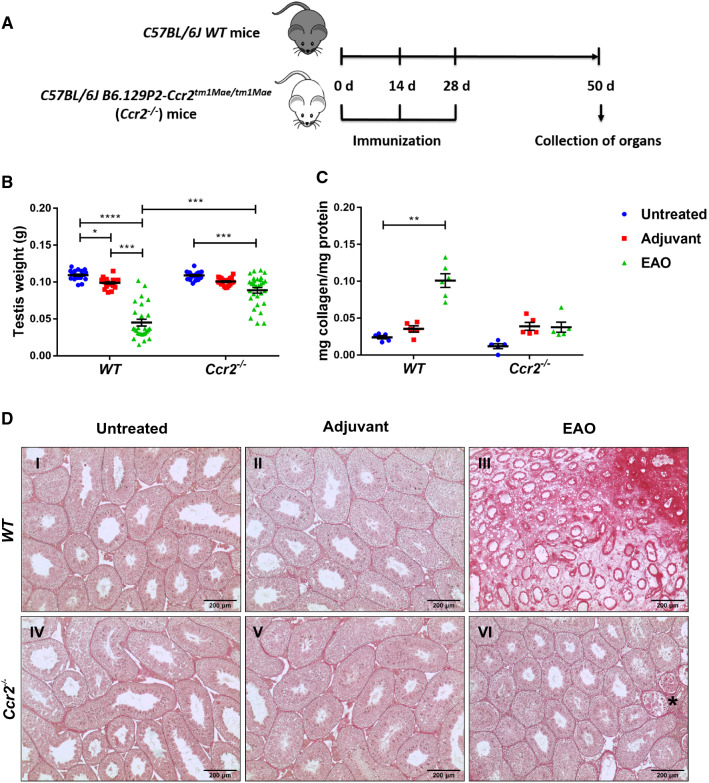
Tissue damage and collagen deposition caused by EAO is reduced in *Ccr2*^*−/−*^ mouse testes. Schematic diagram representing the time points for EAO induction and organ collection in *C57BL/6J* (*WT*) and *B6.129P2-Ccr2*^*tm1Mae/tm1Mae*^ (*Ccr2*^*−/−*^) mice. Mice were immunized three times every other week starting at day 0, and testes were collected at day 50 (**A**). Paired testis weight (**B;** *n* = 20–30) and total collagen content determined by hydroxyproline assay (**C;** *n* = 5–6) in untreated, adjuvant control, and EAO testes from *WT* and *Ccr2*^*−/−*^ mice. Representative photomicrographs of picro-sirius red-stained paraffin sections (**D**) of untreated **(I, IV)**, adjuvant control **(II, V),** and EAO **(III, VI)** testes from *WT* **(I**–**III)** and *Ccr2*^*−/−*^ mice **(IV**–**VI)**; asterisk in (**VI**) indicates seminiferous tubule damage. Values are mean ± SEM; statistical analyses used the Kruskal–Wallis test followed by Dunn’s multiple comparison test. *P < 0.05, **P < 0.01, ***P < 0.001, ****P < 0.0001

Adjuvant control animals (*n* = 22) received CFA mixed with 0.9% NaCl instead of TH, following the same injection regimen. Age-matched untreated mice (*n* = 21) were also included in the study. Animals were sacrificed by cervical dislocation 50 days after the first immunization (following deep anesthesia induced with 5% isoflurane). A schematic diagram illustrating the time course of immunizations and time points for organ collection is shown in Fig. 1A. The testes were removed, weighed, and either snap-frozen in liquid nitrogen or fixed in Bouin’s solution for embedding in paraffin. Freshly collected testes were used for flow cytometric analysis.

### Induction of EAO in mice with elevated follistatin levels

Thirty days before the first immunization with TH, *C57BL/6J* mice were injected intramuscularly with non-replicative recombinant adeno-associated viral (rAAV) vector serotype 6 carrying a gene cassette of the circulating form of follistatin (rAAV-FST315; *n* = 13) or an empty gene cassette (empty vector; EV; *n* = 18), as previously described [21]. Adjuvant-only and non-immunized controls were included. The immunization protocol was modified according to Monash University Animal Ethics committee requirements regarding the use of adjuvants: mice were immunized once with TH in CFA, followed by two further immunizations with TH in incomplete Freund’s adjuvant with *B. pertussis* toxin. Approximately 75% of mice immunized by this protocol developed EAO, ranging from mild to severe, within 50 days. Treatment with rAAV-FST315 resulted in a fivefold increase in serum follistatin levels by the time of the first immunization [21]. After collection, testes were either snap-frozen in liquid nitrogen for RNA analysis or fixed in Bouin’s solution for paraffin embedding and subsequent immunofluorescence [21]. A schematic diagram illustrating the time course of vector injection, immunizations, and time points for organ collection is shown in Fig. 4A.

### Human testicular biopsies

Bouin’s-fixed paraffin-embedded testicular tissue samples obtained by routine open testicular biopsy (using the testicular sperm extraction [TESE] protocol) were provided by the University Hospital Zagreb, (Department of Urology, in cooperation with the School of Medicine) at the University of Zagreb. According to current guidelines, three pieces of tissue were obtained through corresponding incisions of the tunica albuginea (upper and lower testis pole and equatorial plane). Biopsied tissues were divided into two parts to provide an accurate diagnosis: one half for cryopreservation and the other half for histological analysis [34, 35]. This study was performed in line with the principles of the Declaration of Helsinki. Each patient provided written consent for the TESE procedure as well as the histological analysis/current study. Approval was granted by the Ethics Committee of the School of Medicine at the University of Zagreb (380-59-10106-20-111/171). The specimens (selected from infertile men with non-obstructive azoospermia) showed histological diagnoses of focal inflammatory lesions associated with disturbed spermatogenesis (mixed atrophy or Sertoli cell-only phenotype) and fibrotic remodeling (thickening of the lamina propria, interstitial accumulation of collagen; tubular fibrosis; *n* = 5). The control group comprised three patients suffering from obstructive azoospermia with fully preserved testicular parenchyma, revealing normal intact spermatogenesis without inflammatory signs.

### Picro-sirius red staining

Testicular histology and collagen distribution were assessed in paraffin sections by picro-sirius red staining [36].

### Hydroxyproline assay

Total testicular collagen content was quantified by chromogenic determination of hydroxyproline concentration using the QuickZyme Total Collagen Assay Kit (QuickZyme Biosciences, Leiden, Netherlands) according to the manufacturer’s instructions. Total testicular collagen content was normalized to total testicular protein using the QuickZyme Total Protein Assay Kit (QuickZyme Biosciences).

### Isolation and treatment of Sertoli cells

SCs were isolated from 21-day-old *C57BL/6 J* mice (8 animals/isolation) according to an established protocol [24]. Two days after isolation, SCs were treated with 20 mM hypotonic Tris-hydrochloride solution (Sigma-Aldrich) for 2 min to remove germ cell contamination. One day after hypotonic shock, SCs were treated with 50 ng/ml recombinant mouse TNF (PromoCell, Heidelberg, Germany), 250 ng/ml human recombinant follistatin 288 (FST288; purified from HEK-293 cells transfected with a human FST 288 transgene), or a combination of both [37]. After 24 h in culture, Sertoli cell-conditioned medium (SCCM) was collected. The purity of SCs (> 85%) was determined by double staining for SOX9 (SC marker) and αSMA (PTC marker) [24].

### Generation and treatment of BMDMs

Bone marrow progenitor cells were isolated from adult *C57BL/6J* and *Ccr2*^*−/−*^ mice (*n* = 28) according to a published protocol [38] and cultured in a complete RPMI-1640 medium (Gibco). BMDMs were generated by treating the progenitor cells with 50 ng/ml mouse recombinant (M-CSF (Miltenyi Biotec) and then stimulating with 25 or 50 ng/ml human recombinant activin A (Miltenyi Biotec), 250 ng/ml FST288, or a combination of both for 6 days.

For gelatin zymography, after 6 days in culture, BMDMs were treated with 50 ng/ml M-CSF and 50 ng/ml activin A or 250 ng/ml FST288 or a combination of both activin A and FST288 in serum-free RPMI-1640 medium. BMDM-conditioned medium was collected 24 h later.

For SC-conditioned medium (SSCM) treatment, BMDMs were generated as above (50 ng/ml M-CSF for 3 days) and subsequently incubated with SCCM for an additional 3 days.

### Flow cytometry

Testes were decapsulated and digested in phosphate-buffered saline (PBS; Gibco, Bleiswijk, Netherlands) containing 1.2 mg/ml collagenase A (Roche Diagnostic, Mannheim, Germany) and 15 U/ml DNase I (Roche Diagnostic) in a 34 °C water bath for 15 min, with agitation. After the sedimentation of the seminiferous tubules, supernatants containing interstitial cells were collected. BMDMs growing in Nunc UpCell 6-well Multidishes (Thermo Fisher Scientific, Roskilde, Denmark) were detached at room temperature (RT) for 30 min. All incubation steps were performed at 4 °C. A total of 1 × 10^6^ cells were blocked with mouse FcR blocking reagent (Miltenyi Biotec, Bergisch-Gladbach, Germany) for 10 min, followed by incubation with the appropriate antibodies (see Table 1 for details) for 15 min. Afterwards, cells were permeabilized using the Fix/Permeabilization Staining Buffer Set (Miltenyi Biotec) for 30 min and blocked again. Subsequently, the cells were incubated with anti-fibronectin or anti-collagen I antibody for 45 min and the respective secondary antibodies for 30 min at 4 °C. Data were collected using the MACSQuant Analyzer 10 flow cytometer (Miltenyi Biotec) and analyzed using FlowJo V10 software (FlowJo LLC, Oregon, USA). The flow cytometry gating strategy is shown in Supplementary Fig. S1.

**Table 1:** List of antibodies and isotype control antibodies used for flow cytometric analysis

Antibodies	Companies	Catalog No	Clones	Dilution
Recombinant monoclonal anti-CD45 VioGreen REAfinity™	Miltenyi Biotec, Bergisch-Gladbach, Germany	130-110-803	REA737	1:50
Rat monoclonal anti-CD45 PE	BioLegend, San Diego, USA	103106	30-F11	1:100
Rabbit polyclonal anti-fibronectin	Abcam, Cambridge, UK	ab2413	–	1:100
Rabbit polyclonal anti-collagen I, biotin	Rockland, Limerick, USA	600-406-103	–	1:200
Recombinant monoclonal anti-CXCR4 PE REAfinity™	Miltenyi Biotec	130-118-682	REA107	1:50
Rat monoclonal anti-Ly6G APC	BioLegend	127614	1A8	1:100
Rat monoclonal anti-Ly6C PerCP	BioLegend	128027	HK1.4	1:80
Rat monoclonal anti-Ly6C VioGreen	Miltenyi Biotec	130-102-207	1G7.G10	1:50
Recombinant monoclonal anti-CD11c PE-Vio770 REAfinity™	Miltenyi Biotec	130-110-840	REA754	1:50
Recombinant monoclonal anti-F4/80 APC REAfinity™	Miltenyi Biotec	130-116-547	REA126	1:50
Recombinant monoclonal anti-CD11b APC-Vio770 REAfinity™	Miltenyi Biotec	130-113-803	REA592	1:50
Rat monoclonal anti-CD11b PerCP/Cy5.5	BioLegend	101228	M1/70	1:80
Rat monoclonal anti-CD64 PerCP/Cy7	BioLegend	139314	X54-5/7.1	1:40
Rat monoclonal anti-CCR2 FITC	BioLegend	150608	SA203G11	1:50
Goat anti-rabbit Alexa Fluor 488	Invitrogen, Oregon, USA	A-11034	–	1:500
Streptavidin Alexa Fluor 488	BioLegend	405235	–	1:1000
Recombinant monoclonal REA control VioGreen	Miltenyi Biotec	130-104-624	REA293	1:50
Recombinant monoclonal REA control PE	Miltenyi Biotec	130-104-628	REA293	1:50
Recombinant monoclonal REA control PE-Vio770	Miltenyi Biotec	130-104-632	REA293	1:50
Recombinant monoclonal REA control APC	Miltenyi Biotec	130-104-630	REA293	1:50
Recombinant monoclonal REA control APC-Vio770	Miltenyi Biotec	130-104-634	REA293	1:50

### Immunofluorescence

Frozen testicular sections (10 μm) or fresh BMDMs were fixed in ice-cold methanol for 10 min. BMDMs were permeabilized using 0.5 M glycine for 30 min at RT. Subsequently, specimens were blocked in 5% bovine serum albumin (BSA; Sigma-Aldrich, Steinheim, Germany) and 10% goat serum (BioLegend, San Diego, USA) in Tris-buffered saline containing 0.1% Tween-20 (TBST) at RT for 2 h. After incubating with the appropriate primary antibodies overnight (see Table 2 for details), the sections were washed with TBST and incubated with the corresponding secondary antibodies at RT for 1 h. Nuclei were counterstained with Topro 3 (Life Technologies, Darmstadt, Germany). Finally, the slides were mounted in ProLong™ Gold Antifade Mountant containing DAPI (Invitrogen, Oregon, USA). Fluorescence images were captured on a confocal laser scanning microscope 710 (Carl Zeiss, Göttingen, Germany).

**Table 2:** List of primary and secondary antibodies used in immunofluorescence staining (IF) and western blotting (WB)

Antibodies	Company	Catalog No	Dilution
***Primary antibodies***			
Rabbit polyclonal anti-fibronectin; IF	Abcam	ab2413	1:300 (Testis) 1:200 (BMDM)
Rabbit monoclonal anti-collagen I; IF	Abcam	ab21286	1:300 (Frozen) 1:100 (Paraffin)
Rat monoclonal anti-CXCR4 Alexa Fluor 647; IF	BioLegend	146,503	1:100
Rat monoclonal anti-F4/80; IF	Bio-Rad, Munich, Germany	MCA497G	1:200 (Frozen)
Rat monoclonal anti-F4/80; IF	BioLegend	123,101	1:50 (Paraffin)
Mouse monoclonal anti-CD68; IF	BioLegend	375,602	1:20
Rabbit monoclonal anti-CCR2; WB	Abcam	ab203128	1:1000
Mouse monoclonal anti-β-Actin; WB	Sigma-Aldrich, Darmstadt, Germany	A5441	1:5000
Rabbit monoclonal anti-phospho-SMAD2 (Ser465/467); WB	Cell Signaling Technology, Danvers, USA	3108	1:1000
Rabbit monoclonal anti-SMAD2/3 (D7G7) XP; WB	Cell Signaling Technology	8685	1:1000
***Secondary Antibodies***			
Goat anti-rat IgG (H + L) Alexa Fluor 546; IF	Invitrogen	A-11081	1:1500
Goat anti-rabbit IgG (H + L) Alexa Fluor 488; IF	Invitrogen	A-11034	1:1500
Goat anti-mouse IgG F(ab)2 Alexa Fluor 488; IF	Jackson ImmunoResearch Labs, West Grove, USA	115-546-072	1:800
HRP-conjugated sheep anti-mouse IgG (H + L); WB	Sigma-Aldrich	A5906	1:5000
HRP-conjugated goat anti-rabbit IgG (H + L); WB	MP Biomedicals, Eschwege, Germany	0855676	1:1000

Paraffin-embedded testicular sections (8 μm) were deparaffinized in xylene twice for 10 min and rehydrated by immersing in a series of ethanol solutions of gradually decreasing concentrations. Afterwards, sections were treated with pre-warmed proteinase K solution (20 μg/ml; Sigma-Aldrich) for 3 min at RT. Sections were then blocked in 5% BSA and 10% goat serum in TBS at RT for 1.5 h and incubated with primary antibodies in blocking solution at RT for 4 h (see Table 2 for details). Subsequently, sections were washed with TBS and incubated with the corresponding secondary antibodies at RT for 1 h, followed by treatment with 0.3% Sudan Black B (Sigma-Aldrich) for 5 min to reduce autofluorescence. Finally, after several washes with TBS, sections were mounted using ProLong™ Gold Antifade Mountant containing DAPI and photographed using a confocal laser scanning microscope, as above.

### RNA extraction and qRT-PCR

Total RNA was isolated from frozen testes using the RNeasy Fibrous Tissue Mini Kit (Qiagen, Hilden, Germany) and from cells using the RNeasy Mini Kit (Qiagen), according to the manufacturer’s instructions. Contaminating DNA was removed using the RNase-Free DNase Set (Qiagen). Total RNA was reverse transcribed as described previously [21, 24]. Quantitative reverse transcription-polymerase chain reaction (qRT-PCR) was performed using iTaq Universal SYBR Green Supermix (Bio-Rad, Munich, Germany) for self-designed primers or QuantiTect SYBR Green PCR Master Mix (Qiagen) for QuantiTect primer assays (Qiagen) in a CFX96 Touch thermal cycler (Bio-Rad). Primer sequences and annealing temperatures are listed in Table 3. Hypoxanthine–guanine-phosphoribosyltransferase (*Hprt*) and *18S rRNA* were selected as stably expressed housekeeping genes. All samples were run as duplicates. Relative mRNA expression was calculated using the 2^−ΔΔCt^ method [39].

**Table 3:** List of primers used in qRT-PCR (F: forward primer, R: reverse primer)

Gene	Sequence (5′–3′)	Amplicon size (bp)	Annealing temperature (°C)
***18S rRNA***	F: TACCACATCCAAGGAAGGCAGCA	180	55
	R: TGGAATTACCGCGGCTGCTGGCA		
***Acvr1b***	F: CCAACTGGTGGCAGAGTTAT	119	55
	R: CTGGGACAGAGTCTTCTTGATG		
***Acvr2b***	F: ATGAGTACATGCTGCCCTTC	101	55
	R: CTTAATCGTGGGCCTCATCTT		
***Adgre1 *(F4/80)**	F: TCTGCAGTGTCAGCTCAGAA	277	60
	R: GAAGTCTGGGAATGGGAGCT		
***Ccl2***	QuantiTect Primer assay (QT00167832, Qiagen)	118	55
***Cxcl12***	F: CAGTGACGGTAAACCAGTCAGC	68	64
	R: TGGCGATGTGGCTCTCG		
***Cxcr4***	F: GACTGGCATAGTCGGCAATG	131	60
	R: AGAAGGGGAGTGTGATGACAAA		
***Fn1* (fibronectin)**	F: AGAAGGCAGTAGCACAGA	110	55
	R: TCTCCTCCACAGCATAGATAG		
***Fst***	F: AGGAGGATGTGAACGACAATAC	95	55
	R: CACGTTCTCACACGTTTCTTTAC		
***Hprt***	F: CTGGTAAAAGGACCTC	110	55
	R: CTGAAGTACTCATTATAGTCAAG		
***Il-1a***	F: CGAAGACTACAGTTCTGCCATT	126	60
	R: GACGTTTCAGAGGTTCTCAGAG		
***Il-1b***	F: CAACCAACAAGTGATATTCTCCATG	152	60
	R: GATCCACACTCTCCAGCTGCA		
***Il-6***	QuantiTect Primer assay (QT00098875, Qiagen)	128	55
***Il-10***	QuantiTect Primer assay (QT00106169, Qiagen)	109	55
***Inha***	F: GCCAAGGTGAAGGCTCTATT	126	55
	R: AGACCTCCTGTGCATGAAAC		
***Inhba***	F: AGAACGGGTATGTGGAGATAGA	97	55
	R: GACTCGGCAAAGGTGATGAT		
***Inhbb***	F: CTGCCAGTCGGGCAGGGTATAA	110	55
	R: CCTTCACTCCACCAGTCATTT		
***Itgav***	F: AAGGCGCAGAATCAAGGGGA	182	60
	R: CCAGCCTTCATCGGGTTTCC		
***Itgb3***	F: TGGTGCTCAGATGAGACTTTGTC	86	60
	R: GACTCTGGAGCACAATTGTCCTT		
***Itgb5***	F: TGTTCAGCTACACAGAACTGCCCA	198	55
	R: TTTGGAACTTGGCAAACTCTCGGC		
***Mmp2***	F: CAGGGAATGAGTACTGGGTCTATT	119	60
	R: ACTCCAGTTAAAGGCAGCATCTAC		
***Mmp9***	F: AAGGACGGCCTTCTGGCACACGCCTTT	874	60
	R: GTGGTATAGTGGGACACATAGTGG		
***Mmp14***	F: TTACAAGTGACAGGCAAGG	112	60
	R: GCTTCCTCCGAACATTGG		
***Pdgfa***	F: CAAGACCAGGACGGTCATTT	223	55
	R: CCTCACCTGGACCTCTTTCA		
***Pdgfb***	F: CCCACAGTGGCTTTTCATTT	137	55
	R: GTGAACGTAGGGGAAGTGGA		
***Pdgfrb***	F: GAACGACCATGGCGATGAGA	146	60
	R: GCATCGGATAAGCCTCGAACA		
***Timp1***	F: CCCCAGAAATCAACGAGAC	156	55
	R: CTGGGACTTGTGGGCATATC		
***Tnf***	QuantiTect Primer assay (QT00104006, Qiagen)	112	55

### Bulk RNA sequencing

Total RNA was isolated from BMDMs using the RNeasy Mini Kit (Qiagen) combined with on-column DNase digestion (RNase-Free DNase Set, Qiagen) to avoid contamination by genomic DNA. RNA and library preparation integrity were verified with the LabChip Gx Touch 24 (Perkin Elmer, Waltham, USA). Library preparation was carried out with 4 µg of total RNA as input using the VAHTS Stranded mRNA-seq Library Prep kit according to the manufacturer’s protocol (Vazyme, Nanjing, China). Sequencing was performed on the NextSeq500 System (Illumina, San Diego, USA) using v2 chemistry with a 1 × 75 bp single-end setup. The resulting raw reads were assessed for quality, adapter content, and duplication rates using FastQC [40]. Trimmomatic version 0.39 was employed to trim reads after a quality drop below a mean of Q20 in a window of 5 nucleotides [41]. Only reads between 30 and 150 nucleotides were cleared for further analysis. Trimmed and filtered reads were aligned against Ensembl mouse genome version mm10 (ensemble release 101) using STAR 2.7.9a with the parameter “–outFilterMismatchNoverLmax 0.1” to increase the maximum ratio of mismatches to mapped length to 10% [42]. The number of reads aligning to genes was counted using the featureCounts 2.0.2 tool of the Subread package [43]. Only reads mapping at least partially inside exons were admitted and aggregated per gene; reads overlapping multiple genes or aligning to multiple regions were excluded. Differentially expressed genes (DEGs) were identified using DESeq2 version 1.30.0 [44]. Only genes with a minimum fold change of ± 2 (log2 =  ± 1), a maximum Benjamini-Hochberg-corrected p-value of 0.05, and a minimum combined mean of five reads were deemed to be significantly differentially expressed. Ensemble annotation was enriched using UniProt data (release 06.06.2014) based on Ensembl gene identifiers [45].

### Protein extraction and Western blot

BMDMs were lysed on ice in RIPA buffer (150 mM NaCl, 0.1% Triton X-100, 0.5% sodium deoxycholate, 0.1% sodium dodecyl sulfate [SDS], 50 mM Tris–HCl pH 8.0, 2 mM EDTA) supplemented with protease inhibitor cocktail (1:100; Sigma-Aldrich) and centrifuged at 13,000×*g* for 30 min at 4 °C. The RC DC Protein Assay (Bio-Rad, Hercules, USA) was used to measure total protein concentration. Protein samples diluted in Laemmli buffer (30 µg/lane) were resolved by 10% (SMADs only) or 12.5% SDS–polyacrylamide gel electrophoresis, transferred onto nitrocellulose membrane (GE Healthcare, Darmstadt, Germany), blocked with 5% non-fat milk in TBST for 1 h at RT, and subsequently washed in TBST. Incubation with primary antibodies was performed overnight at 4 °C (Table 2). Afterwards, membranes were washed three times for 10 min at RT in TBST before incubation with the appropriate secondary antibodies for 1 h at RT (Table 2). Finally, the membranes were probed with SuperSignal West Pico Chemiluminescent Substrate (Thermo Fisher Scientific, Waltham, MA, USA) and subsequently developed using the Fusion FX Western Blot and chemiluminescence imaging system (Vilber Lourmat, Eberhardzell, Germany). The results were quantified using FusionCapt Advance Solo 4 16.07 software (Vilber Lourmat).

### BMDM migration assay

BMDM generation and treatment with activin A and/or FST288 were performed as described above, and cells were cultivated in a Nunc UpCell 6-well dish before harvesting. For the migration assay, cells were gently washed with PBS and incubated on ice for 15 min before collection. After seeding the BMDMs (3 × 10^5^) in Transwell inserts (8 µm pore size; Corning Life Sciences, Tewksbury, USA) in serum-free RPMI-1640 medium, 750 µl serum-free RPMI-1640 medium containing 500 ng/ml CCL2 was added to the lower chamber. Cells were allowed to migrate for 3 h at 37 °C. Migrated cells were collected from the lower chamber and counted using the MACSQuant Analyzer 10 flow cytometer (Miltenyi Biotec).

### Gelatin zymography

The enzymatic activity levels of MMP2 and MMP9 were measured in BMDM-conditioned medium (generated as described above); gelatin zymography was performed as reported [46]. BMDM-conditioned medium was loaded and gels were stained with Coomassie Brilliant blue solution. Band intensity was analyzed using ImageJ software (National Institutes of Health, Bethesda, USA). Relative enzymatic activity was calculated by normalizing the band intensity of the untreated group to 1.0.

### Statistical analysis

Statistical data analysis was performed using GraphPad Prism 7 software (GraphPad Software, San Diego, USA). Data are presented as mean ± standard error of the mean (SEM) and were tested for normal distribution using the Kolmogorov–Smirnov test. The statistical significances of differences in multiple groups were calculated using one-way or two-way analysis of variance (ANOVA) followed by Bonferroni’s multiple comparisons test (Gaussian distribution) or the Kruskal–Wallis test followed by Dunn’s multiple comparisons test (non-normal distribution). Correlation significance between two groups was analyzed using the Pearson coefficient (normal distribution) or Spearman coefficient (non-normal distribution). Statistical significance was set at *P* < 0.05.

## Results

### *Ccr2* deficiency protects the testis from damage caused by EAO

To determine the role of CCR2 in EAO-induced fibrosis, we compared EAO in *WT* and *Ccr2*^*−/−*^ mice. Measurement of testicular weight 50 days after the first immunization showed that EAO caused a reduction in mean testis weight in both *WT* and *Ccr2*^*−/−*^ mouse strains, compared with untreated and adjuvant control groups (Fig. 1B). However, mean testis weight was significantly higher in the *Ccr2*^*−/−*^ EAO mice (0.089 ± 0.004 g) than in the *WT* EAO mice (0.045 ± 0.004 g) (Fig. 1B), indicating lower levels of testicular damage in the *Ccr2* null mice. To quantify the fibrotic response, testicular collagen content was analyzed by hydroxyproline assay and picro-sirius red staining and found to be significantly elevated in *WT* EAO testes, compared with *WT* untreated testes (Fig. 1C). In contrast, EAO did not result in significantly increased levels of collagen in *Ccr2*^*−/−*^ mice, and total collagen content was 2.7-fold lower in EAO testes than in *WT* testes (Fig. 1C). Histological examination of testicular sections revealed that normal testicular architecture was destroyed in 94% of *WT* EAO testes, with germ cell sloughing, atrophy of seminiferous tubules, thickening of the lamina propria of seminiferous tubules, and an increase in the density of collagen fibers (Fig. 1D). In contrast, although a few disrupted seminiferous tubules were visible in 3 of 15 mice (20%), testicular structure was largely intact in *Ccr2*^*−/−*^ EAO testes, indicating a crucial role for CCR2 in inflammation and damage following EAO (Fig. 1D).

### *Ccr2* deficiency reduces inflammatory responses in EAO testes

**Fig. 2:**
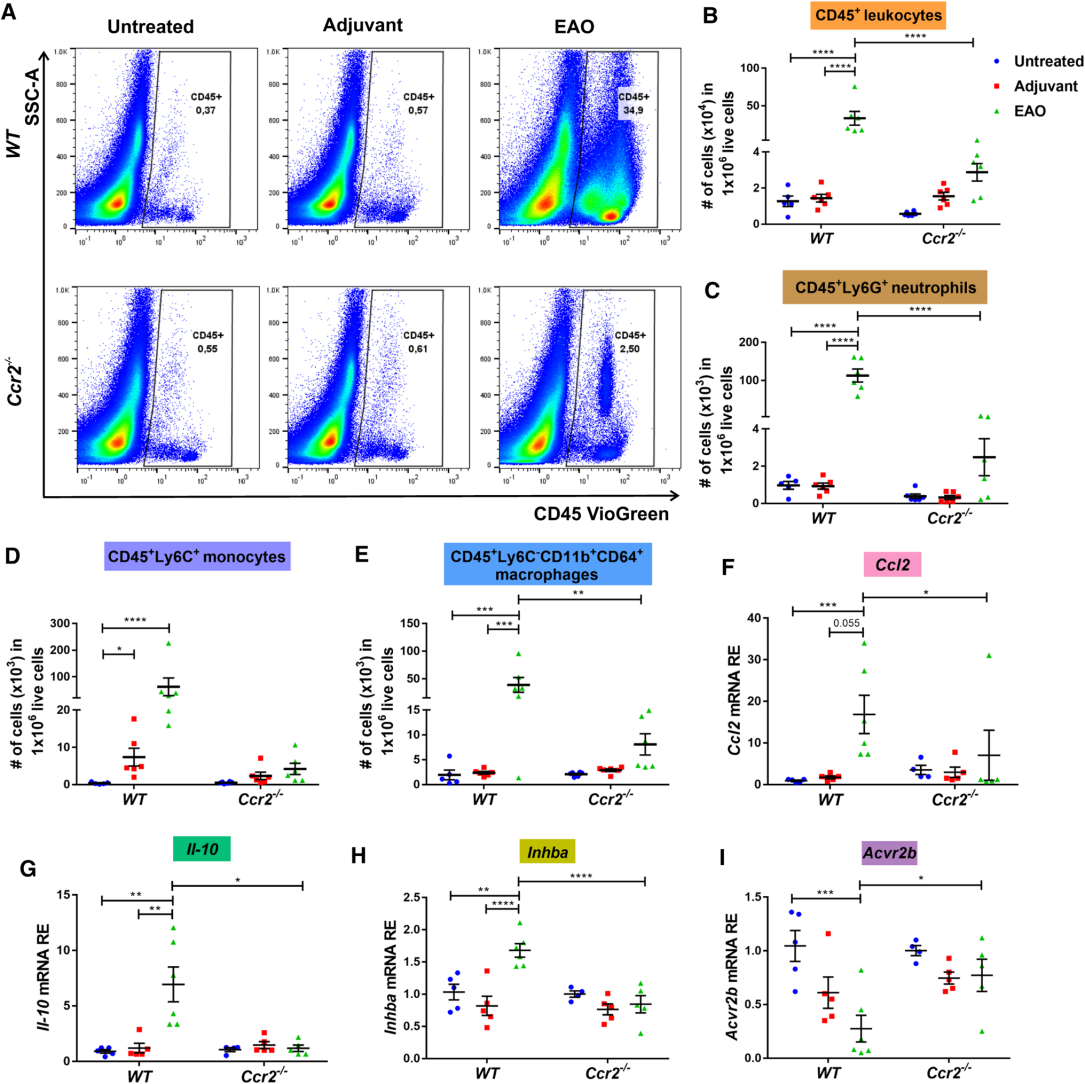
Immune cell infiltration and expression of inflammatory mediators caused by EAO are reduced in *Ccr2*^*−/−*^ mouse testes. Representative density plots for CD45^+^ leukocytes in untreated, adjuvant control, and EAO testicular single-cell suspensions from *WT* and *Ccr2*^*−/−*^ mice (**A**). After gating out doublets and nonviable cells, numbers of testicular CD45^+^ leukocytes (**B**), CD45^+^Ly6G^+^ neutrophils (**C**), CD45^+^Ly6C^+^ monocytes (**D**), and CD45^+^Ly6C^−^CD11b^+^CD64^+^ macrophages (**E**), were determined in untreated, adjuvant control, and EAO testicular single-cell suspensions from *WT* and *Ccr2*^*−/−*^ mice by flow cytometry (*n* = 5–6). Relative expression (RE) of *Ccl2* (**F**), *Il-10* (**G**), *Inhba* (**H**), and *Acvr2b* **(I)** was determined in untreated, adjuvant control, and EAO testes from *WT* and *Ccr2*^*−/−*^ mice by qRT-PCR analysis and normalization to *18S rRNA* and *Hprt* (*n* = 4–6). Values are mean ± SEM; statistical analyses used the Kruskal–Wallis test followed by Dunn’s multiple comparison test or two-way ANOVA followed by Bonferroni’s multiple comparison test. *P < 0.05, **P < 0.01, ***P < 0.001, ****P < 0.0001

Increased numbers of CD45^+^ leukocytes and elevation of inflammatory cytokines such as CCL2, TNF, IL-1 and IL-10, and activin A are important hallmarks of the inflamed testis in EAO [6, 13]. To investigate whether depletion of *Ccr2* affects the testicular inflammatory response, the presence and number of immune cells and expression of cytokines and activins in testes were analyzed. Flow cytometry demonstrated significantly lower numbers of total CD45^+^ leukocytes (Fig. 2A and B), CD45^+^Ly6G^+^ neutrophils (Fig. 2C), and CD45^+^Ly6C^−^CD11b^+^CD64^+^ macrophages (Fig. 2E), as well as an approximately 14-fold reduced population of CD45^+^Ly6C^+^ monocytes (Fig. 2D) in *Ccr2*-deficient EAO testes, compared with EAO *WT* testes. These results imply that the depletion of CCR2 inhibits the recruitment of infiltrating immune cells in the EAO testis.

Quantitative RT-PCR showed that *Ccr2* deficiency led to significant suppression of testicular *Ccl2* (Fig. 2F), *Il-10* (Fig. 2G), and *Il-1a* (Supplementary Fig. S2A) mRNAs during EAO, compared with the levels observed in *WT* mice undergoing EAO. Moreover, levels of *Ccl2* and *Il-10* mRNA were not significantly changed in any *Ccr2*^*−/−*^ experimental group (Fig. 2F and G). Absence of *Ccr2* also inhibited the EAO-mediated increase in expression of the activin A subunit *Inhba* (Fig. 2H). Expression of follistatin (*Fst)* was significantly increased in EAO testes (Supplementary Fig. S2E), while expression of the activin A receptor subunit *Acvr1b* was reduced (Supplementary Fig. S2F). In addition, following EAO, expression of the *Acvr2b* subunit was significantly reduced in *WT* testes but remained in the normal range in *Ccr2*^*−/−*^ testes (Fig. 2I). However, other inflammation-related genes, including *Il-1b* and other activin-related genes (*Inhbb* and *Inha*), were not significantly altered in untreated, adjuvant control, or EAO testes from *WT* or *Ccr2*^*−/−*^ mice (Supplementary Fig. S2B and G-H). Furthermore, expression of *Tnf* and *Il-6* mRNA was significantly increased in EAO *WT* testes, but unchanged in all *Ccr2*^*−/−*^ groups (Supplementary Fig. S2C and D).

### *Ccr2* deficiency suppresses changes in the numbers of ECM-expressing immune cells and CXCL12/CXCR4 expression induced by EAO

**Fig. 3:**
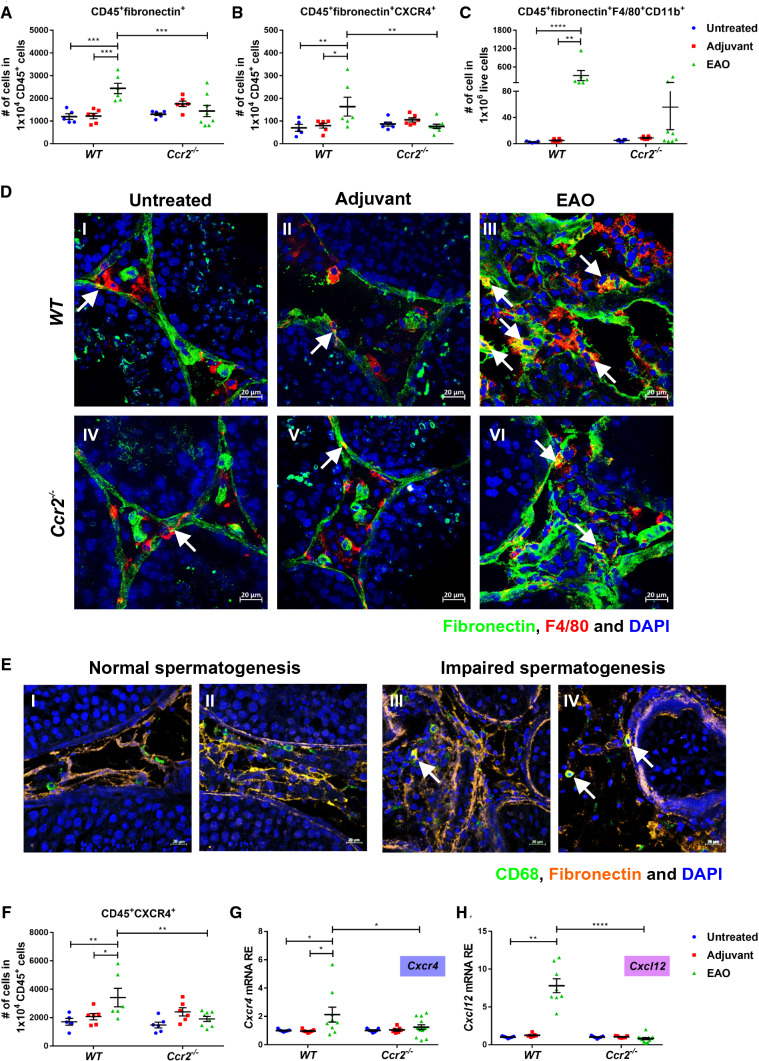
The number of fibronectin^+^ immune cells is increased in mouse testes with EAO and human testes with impaired spermatogenesis. After gating out doublets and nonviable cells, the numbers of testicular CD45^+^fibronectin^+^ cells (**A**), CD45^+^fibronectin^+^CXCR4^+^ cells (**B**), and CD45^+^CXCR4^+^ cells (**F**) within the CD45^+^ leukocyte population and the number of testicular CD45^+^fibronectin^+^F4/80^+^CD11b^+^ cells (**C**) within the live cell population were analyzed in untreated, adjuvant control, and EAO testicular single-cell suspensions from *WT* and *Ccr2*^*−/−*^ mice by flow cytometry (*n* = 5–8). Representative photomicrographs of fibronectin (green), F4/80 (red), and DAPI (blue) triple immunofluorescence staining in frozen sections (**D**) from untreated **(I, IV)**, adjuvant control **(II, V)** and EAO **(III, VI)** testes in *WT* **(I**–**III)** and *Ccr2*^*−/−*^ mice **(IV**–**VI)**; arrows indicate fibronectin^+^ macrophages. Representative photomicrographs of CD68 (green), fibronectin (orange), and DAPI (blue) triple immunofluorescence staining in paraffin sections from human testicular biopsies with normal and impaired spermatogenesis (**E**); arrows indicate triple-positive cells. Relative expression (RE) of *Cxcr4* (**G**) and *Cxcl12* (**H**) in untreated, adjuvant control, and EAO testes from *WT* and *Ccr2*^*−/−*^ mice was determined by qRT-PCR analysis and normalization to *18S rRNA* and *Hprt* (*n* = 7–13). Values are mean ± SEM; statistical analyses used the Kruskal–Wallis test followed by Dunn’s multiple comparison test or two-way ANOVA followed by Bonferroni’s multiple comparison test. *P < 0.05, **P < 0.01, ***P < 0.001, ****P < 0.0001

Since the above results indicated that loss of *Ccr2* expression reduced immune cell accumulation and inhibited the fibrotic response in EAO testes, we wanted to investigate the involvement of immune cells (particularly macrophages) in the development of testicular fibrotic remodeling. Therefore, the numbers and localization of ECM-expressing immune cells in the testes during EAO were analyzed by flow cytometry and immunofluorescence, respectively; CXCR4^+^ cells were also quantified as CXCR4 plays a significant role in ECM-producing immune cells [47]. The proportions of CD45^+^fibronectin^+^ cells (Fig. 3A) and CD45^+^fibronectin^+^CXCR4^+^ cells (Fig. 3B) within the total CD45^+^ leukocyte population were significantly increased in *WT* EAO testes, while *Ccr2* deficiency inhibited this effect. Similar results were observed for CD45^+^collagen I^+^ cells and CD45^+^collagen I^+^CXCR4^+^ cells (Supplementary Fig. S3A and B). Among immune cells, macrophages are an important source of ECM proteins [48]. The numbers of fibronectin^+^ and collagen I^+^ macrophages (identified by F4/80 and CD11b) were increased 117-fold and 70-fold, respectively, in *WT* EAO testes, compared with testes from untreated and adjuvant controls (Fig. 3C and Supplementary Fig. S3C). These results were confirmed by immunofluorescence (Fig. 3D and Supplementary Fig. S3D). In contrast, the numbers of fibronectin- and collagen I-expressing TMs detected in *Ccr2*^*−/−*^ EAO testes were approximately sevenfold lower, compared with the numbers in *WT* EAO testes (Fig. 3C, D and Supplementary Fig. S3C, D). Furthermore, analysis of testicular biopsies from patients with impaired spermatogenesis, testicular fibrosis, and inflammation showed that fibronectin expression was increased in CD68^+^ macrophages. This indicated that similar to mouse EAO, macrophages express fibronectin under pathological conditions (Fig. 3E). These results suggest that infiltrating bone marrow CCR2^+^ cells play a role in modulating the levels of ECM proteins during testicular inflammation and thus also in fibrotic remodeling.

Because CXCR4 and its ligand CXCL12 contribute to the recruitment of circulating progenitors of active fibroblasts [49], we also analyzed the testicular expression of CXCR4 by flow cytometry, qRT-PCR, and immunofluorescence in *WT* and *Ccr2*^*−/−*^ EAO mouse models. While increased numbers of CD45^+^CXCR4^+^ cells (within the total CD45^+^ leukocyte population) were detected in *WT* testes in response to EAO, this increase was abrogated in the absence of *Ccr2* expression (Fig. 3F). Furthermore, reduced levels of *Cxcr4* (Fig. 3G) and *Cxcl12* (Fig. 3H) mRNA were detected in *Ccr2*^*−/−*^ EAO testes, compared with *WT* EAO testes. Immunofluorescence revealed elevated numbers of CXCR4^+^ macrophages (as well as total numbers of CXCR4^+^ cells) in *WT* EAO testes, while infiltration by CXCR4^+^ macrophages was not increased in *Ccr2*^*−/−*^ EAO testes (Supplementary Fig. S3E). These data suggest that CCR2 signaling is also involved in the recruitment of CXCR4^+^ cells in EAO testis.

### Activin A contributes to the production of collagen I by macrophages and increases MMP expression during EAO

**Fig. 4:**
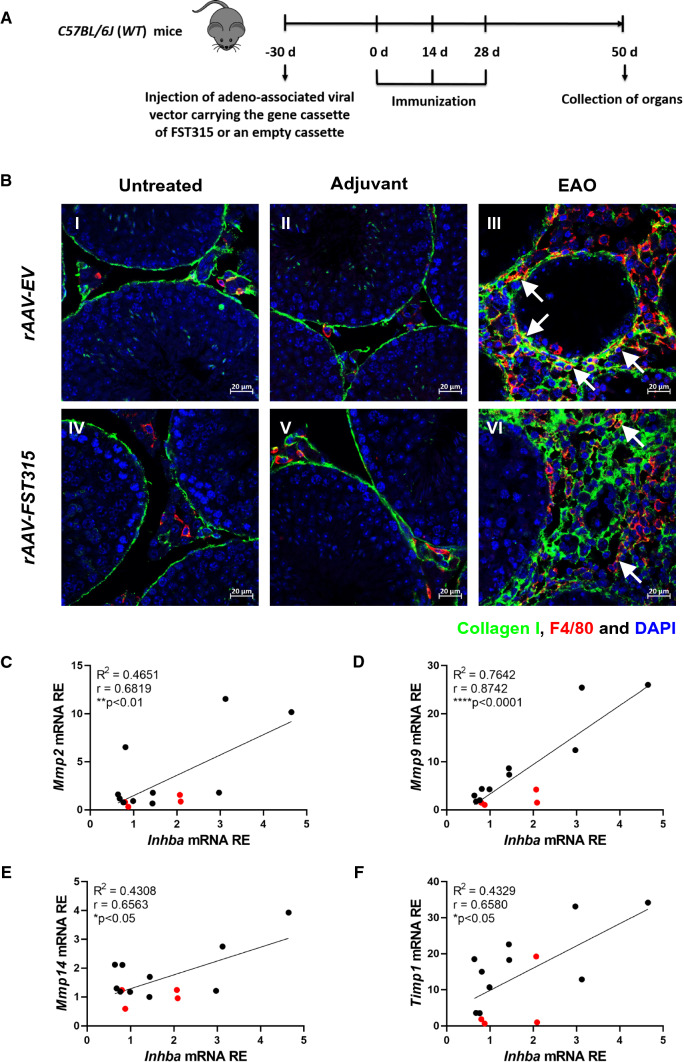
Inhibition of activin A by FST315 reduces collagen I production by macrophages and expression of *Mmps* in EAO testes. Schematic diagram representing the time points for rAAV (adeno-associated viral vector) injection, EAO induction, and organ collection in *C57BL/6 J* (*WT*) mice. Mice were injected with rAAV carrying the FST315 gene cassette (*rAAV-FST315*) or an empty vector (*rAAV-EV*) 30 days before the first EAO immunization. Subsequently, mice were immunized three times every other week starting at day 0, and testes were collected at day 50 (**A**). Representative photomicrographs of collagen I (green), F4/80 (red), and DAPI (blue) triple immunofluorescence staining (**B**) in paraffin sections from untreated **(I, IV)**, adjuvant control **(II, V),** and EAO **(III, VI)** testes in *rAAV-EV* **(I**–**III)** and *rAAV-FST315* **(IV**–**VI)** mice**;** arrows indicate triple-positive cells. The correlations between *Inhba* and *Mmp2* (**C**), *Mmp9* (**D**), *Mmp14* (**E**), and *Timp1* (**F**) relative expression (RE) were determined in *rAAV-EV* and *rAAV-FST315* EAO testes (*n* = 14); red dots represent immunized mice that did not develop EAO. Correlation significance was determined using the Pearson coefficient. *P < 0.05, **P < 0.01, ****P < 0.0001

We previously reported a positive correlation between the severity of disease and testicular activin A concentration [13, 21]. In addition, inhibition of activin A through overexpression of follistatin (using a viral vector expressing FST315) significantly reduced the fibrotic response in EAO [21, 24]. In the present study, the activation of *Inhba* during EAO was suppressed in *Ccr2*-deficient mice (see Fig. 2H), accompanied by decreased macrophage infiltration. Transfection with *FST315* also clearly reduced testicular infiltration by collagen I^+^ TMs in mice undergoing EAO (compared with empty vector [*EV*]-treated control mice; Fig. 4A, B). Collagen I was almost undetectable in TMs from untreated and adjuvant controls (Fig. 4B). These data suggest that activin A is involved in regulating the expression of collagen I by TMs in EAO testis. In addition, as MMPs and their inhibitors TIMPs are key regulators of ECM component degradation and fibrotic remodeling [50], we quantified the levels of *Mmp* and *Timp1* mRNAs by qRT-PCR. Subsequently, correlation analysis demonstrated significant associations between *Inhba* and *Mmp2*, *Mmp9*, *Mmp14,* and *Timp1* mRNA levels in testes from mice treated with control *EV* or *FST315* after induction of EAO (Fig. 4C–F). Altogether, these data suggest that activin A influences the expression of MMPs and TIMP1 by macrophages.

### Activin A affects BMDMs and their transcriptome

**Fig. 5:**
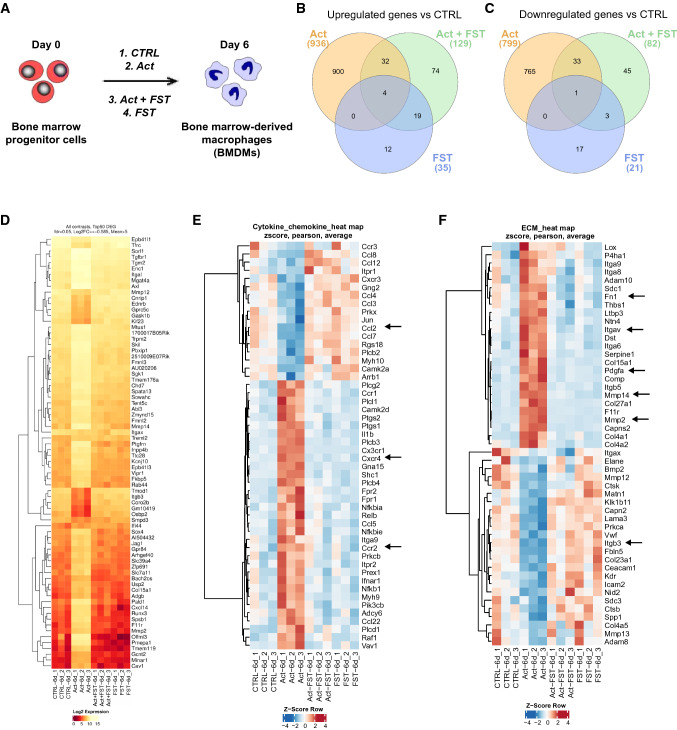
Whole transcriptome analysis of BMDMs treated with activin A in vitro. Experimental set-up for transcriptomic analysis in BMDMs. Cells were left untreated (CTRL) or stimulated with 50 ng/ml activin A (Act), 250 ng/ml FST288 (FST), or a combination of both (Act + FST) (**A;** *n* = 3**)**. Venn diagram of up- (**B**) and downregulated (**C**) DEGs in BMDMs treated with Act, Act + FST, or FST versus CTRL cells. Heat map (**D**) showing expression of the top 50 DEGs in CTRL, Act, Act + FST, and FST groups (based on false discovery rate > 0.05 and minimal count number > 5). Heat maps showing significant DEGs enriched for the terms “Inflammation mediated by chemokine and cytokine signaling pathway” (**E**) and “Extracellular matrix organization” (**F**) from the Panther and Reactome databases, respectively in CTRL, Act, Act + FST, and FST groups of BMDMs, presented with Z-score normalization. Arrows indicate the investigated genes

To investigate how regulation of macrophage function by activin A may contribute to inflammatory and fibrotic responses, mouse BMDMs were stimulated with activin A in vitro (Fig. 5A). FST288 was used as an antagonist of activin A. Phosphorylation of SMAD2 was analyzed to confirm the effect of activin A in BMDMs as activin A downstream signaling is induced by phosphorylation of SMAD2/3 and translocation of SMAD2/3-SMAD4 to the nucleus [22, 24]. Western blotting demonstrated that SMAD2 in BMDMs was phosphorylated within 30 min and 60 min of treatment with 25 ng or 50 ng/ml activin A; these effects were abolished by the addition of 250 ng/ml FST288 (Supplementary Fig. S4A and B).

Next, a whole transcriptomic analysis of BMDMs was performed after treatment with activin A, FST288, or a combination of both (Fig. 5A). Overall, the samples from untreated (CTRL), FST288-alone or FST288 + activin A-treated BMDMs showed similar gene expression patterns, which differed from those identified in activin A-treated BMDMs (Fig. 5B–D). Comparison of the activin A-stimulated BMDMs and the CTRL group revealed 936 upregulated and 799 downregulated DEGs (Fig. 5B and C). Further analysis of the top 50 DEGs identified by the term “inflammation mediated by chemokine and cytokine signaling pathway” from the Panther database demonstrated that several cytokine and chemokine signaling-related genes were regulated by activin A. For example, *Ccr2* and *Cxcr4* were upregulated, while *Ccl2* was downregulated following activin A treatment (Fig. 5E), pointing to dual pro- and anti-inflammatory effects mediated by activin A. In addition, the selection of the top 50 significantly altered DEGs enriched for the term “extracellular matrix organization” from the Reactome database indicated that many upregulated genes, including *Tgfbr1, Fn1*, *Mmp2*, *Mmp14*, *Pdgfa,* and *Itgav*, were related to TGF-β signaling and ECM organization (Fig. 5F). Therefore, we focused on the selected genes (*Ccr2, Cxcr4, Fn1, Mmp2*, *Mmp14*, *Pdgfa, Itgav, and Itgb3*) in the subsequent experiments.

### Activin A upregulates CCR2 in BMDMs and induces the migration of macrophages along a CCL2 gradient

**Fig. 6:**
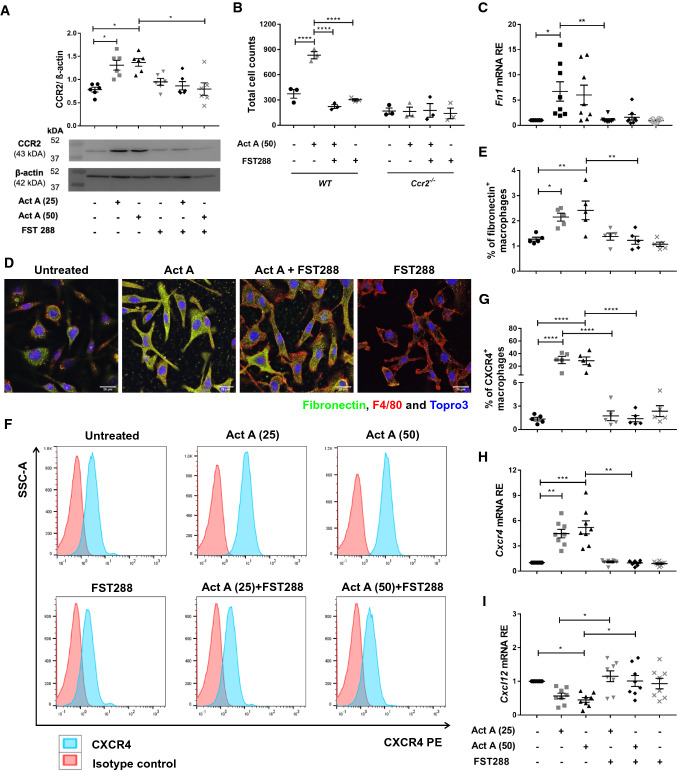
Levels of CCR2, fibronectin, and CXCR4 are elevated in BMDMs following exposure to activin A. Western blotting analysis and quantification of CCR2 protein in BMDMs 6 days after treatment with 25 ng/50 ng/ml activin A, 250 ng/ml FST288, or a combination of both (**A**); β-actin was used as a loading control (*n* = 6). Migration of BMDMs generated from *WT* and *Ccr2*^*−/−*^ mice in the presence of 50 ng/ml activin A, 250 ng/ml FST288, or both was quantified by incubating within a 500 ng/ml CCL2 gradient in a transwell system (**B**); migrated cells in the lower chamber were quantified by flow cytometry (*n* = 3). Relative expression (RE) of *Fn1* (**C**), *Cxcr4* (**H**), and *Cxcl12* (**I**) in BMDMs 6 days after treatment with 25 ng/50 ng/ml activin A, 250 ng/ml FST288, or a combination of both was determined by qRT-PCR analysis and normalization to *Hprt* (*n* = 8). Fibronectin (green), F4/80 (red), and DNA (Topro3; blue) immunofluorescence staining in BMDMs left untreated or treated with 50 ng/ml activin A, 250 ng/ml FST288, or a combination of both (**D**). After gating CD45^+^CD11c^−^Ly6C^−^F4/80^+^CD11b^+^ macrophages, the percentages of fibronectin^+^ (**E**) and CXCR4^+^ (**F**, **G**) macrophages in single-cell BMDM suspensions following treatment with 25 ng/50 ng/ml activin A, 250 ng/ml FST288, or a combination of both were measured by flow cytometry (*n* = 5). Values are mean ± SEM; statistical analyses used the Kruskal–Wallis test followed by Dunn’s multiple comparison test or one-way/two-way ANOVA followed by Bonferroni’s multiple comparison test. *P < 0.05, **P < 0.01, ***P < 0.001, ****P < 0.0001

The above RNAseq data analysis indicated that treating BMDMs with 50 ng/ml activin A-induced elevated *Ccr2* expression. This was confirmed by Western blotting (Fig. 6A), while the addition of FST288 abolished this increase at both mRNA and protein levels (Figs. 5E and 6A). To investigate these findings at a functional level, we analyzed the ability of BMDMs (isolated from *WT* and *Ccr2*^*−/−*^ mice in the absence/presence of activin A and/or FST288) to migrate along a CCL2 gradient. Given that CCL2 stimulates macrophage migration through binding to CCR2 [51], increasing the levels of CCR2 was anticipated to increase migratory capacity. Accordingly, the results of transwell migration assays revealed that activin A significantly increased migration by *WT* BMDMs, while this effect was abolished by the addition of FST288 (Fig. 6B). In contrast, activin A did not influence the migratory capability of *Ccr2*^*−/−*^ BMDMs (Fig. 6B).

### Activin A increases fibronectin and CXCR4 expression in BMDMs

In vivo analyses demonstrated that numbers of both fibronectin^+^ and CXCR4^+^ macrophages were elevated in fibrotic lesions during EAO (Fig. 3 and Supplementary Fig. S3). Moreover, RNAseq confirmed that fibronectin (*Fn1)* and *Cxcr4* were upregulated upon activin A stimulation of BMDMs (Fig. 5E and F). Consistent with these findings, qRT-PCR and immunofluorescence showed that fibronectin mRNA and protein levels were increased in BMDMs after activin A treatment (Fig. 6C and D); these effects were inhibited by FST288. Activin A treatment also induced changes in the morphology of BMDMs from round, oval, or irregular to more elongated in shape (Fig. 6D); this was accompanied by a decrease in the mean fluorescence intensity of the macrophage-specific marker F4/80 (Supplementary Fig. S4C and D). In addition, as TNF stimulates activin A secretion by mouse SCs in vitro, culture medium from TNF-stimulated SCs (SCCM) was used to evaluate the effects on macrophages of activin A originating directly from testicular cells [24]. Analysis by qRT-PCR confirmed that TNF-stimulated SCCM induced a significant increase in *Fn1* mRNA expression in BMDMs (Supplementary Fig. S5A), while non-TNF-SCCM had no effect. FST288 inhibited the increase in *Fn1* mRNA, confirming that the effect of SCCM was due to activin A (Supplementary Fig. S5A). Moreover, flow cytometry showed that activin A treatment of BMDMs significantly increased the percentage of fibronectin^+^ macrophages (Fig. 6E), while the percentage of fibronectin^+^ monocytes was significantly decreased in a dose-dependent manner (Supplementary Fig. S4E).

Similarly, activin A treatment caused a fivefold increase in *Cxcr4* mRNA expression in cultured BMDMs; increased percentages of CXCR4^+^ macrophages (approximately 22-fold) and monocytes (approximately fourfold) were also observed (Fig. 6F–H and Supplementary Fig. S4F). In contrast, the mRNA expression of the CXCR4 ligand Cxcl12 was reduced in activin A-treated BMDMs (Fig. 6I). Moreover, although TNF-stimulated SCCM did not induce increased *Cxcr4* expression in BMDMs, the inclusion of FST288 resulted in reduced levels of *Cxcr4* mRNA (Supplementary Fig. S5B). These data clearly demonstrate the pro-fibrotic function of activin A in BMDMs.

### The expression of MMPs that are dysregulated during EAO is affected by activin A in BMDM culture

**Fig. 7:**
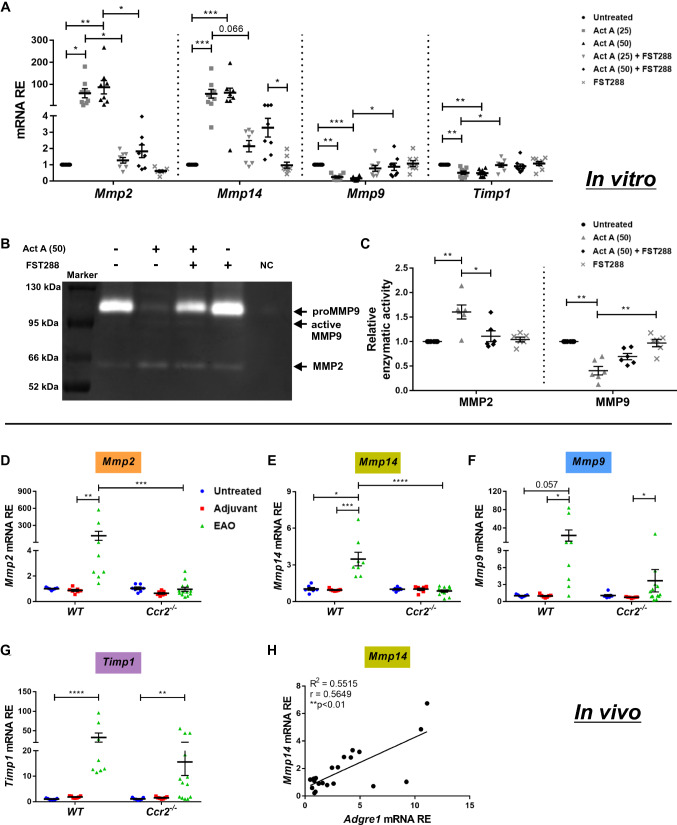
Modulation of MMPs and TIMP1 by activin A in BMDMs in vitro and EAO testes in vivo. Relative expression (RE) of *Mmp2*, *Mmp14*, *Mmp9,* and *Timp1* in BMDMs 6 days after treatment with 25 ng/50 ng/ml activin A, 250 ng/ml FST288, or a combination of both was determined by qRT-PCR analysis and normalization to *Hprt* (**A**; *n* = 8). Representative gelatin zymography gel of BMDMs 7 days after treatment with 50 ng/ml activin A, 250 ng/ml FST288, or a combination of both (**B**); serum-free RPMI-1640 medium was used as a control. Six independent gelatin-zymography replicates were performed and the relative enzymatic activities of MMP2 and MMP9 were calculated by quantifying band intensity (**C**). RE of *Mmp2* (**D**), *Mmp14* (**E**), *Mmp9* (**F**), and *Timp1* (**G**) in untreated, adjuvant control, and EAO testes from *WT* and *Ccr2*^*−/−*^ mice was determined by qRT-PCR and normalization to *18S rRNA* and *Hprt* (*n* = 7–13). The correlation between *Adgre1* and *Mmp14* RE was determined in *WT* and *Ccr2*^*−/−*^ EAO testes (**H**; *n* = 21**)**. Values are mean ± SEM. Statistical analyses used the Kruskal–Wallis test followed by Bonferroni’s multiple comparison test or one-way/two-way ANOVA followed by Tukey’s multiple comparison test; correlation significance was analyzed using Spearman’s coefficient. *P < 0.05, **P < 0.01, ***P < 0.001, ****P < 0.0001

As shown above, increased *Inhba* expression correlated with elevated levels of *Mmp* and *Timp1* mRNAs in EAO testes in vivo (Fig. 4C–F). Moreover, RNAseq indicated that expression of *Mmp2* and *Mmp14* was increased in activin A-stimulated BMDMs (Fig. 5F). Therefore, we investigated the effects of activin A stimulation and *Ccr2* absence on MMPs in vitro and in vivo. Analysis by qRT-PCR showed that activin A significantly upregulated the expression of *Mmp2* and *Mmp14* in BMDMs, while *Mmp9* and *Timp1* mRNAs were downregulated (Fig. 7A); these effects were blocked by FST288. Treatment with TNF-stimulated SCCM also led to a 50-fold increase in *Mmp2* expression (Supplementary Fig. S5C). Notably, treatment of BMDMs with TNF-stimulated SCCM also upregulated the expression of *Mmp14* (46-fold), *Mmp9* (16-fold), and *Timp1* (1.5-fold); however, these increases were not abolished by FST288, pointing to regulation by SC-derived factors other than activin A (Supplementary Fig. S5D–F). In agreement with the qRT-PCR results, gelatin zymography showed that activin A-induced significant upregulation of MMP2 and downregulation of MMP9 enzymatic activity (Fig. 7B and C). However, in contrast, induction of EAO resulted in increased expression of *Mmp9* and *Timp1* in addition to *Mmp2* and *Mmp14* in *WT* testes (Fig. 7D–G). Moreover, the EAO-induced increases in *Mmp2* and *Mmp14* mRNA were significantly reduced in *Ccr2*^*−/−*^ testes (Fig. 7D and E). Although *Mmp9* (Fig. 7F) was also downregulated, this decrease was not statistically significant due to the variable levels of *Mmp9* expression in *Ccr2*^*−/−*^ EAO testes. Our results also indicated that *Timp1* expression was not influenced by *Ccr2* deficiency (Fig. 7G). Interestingly, Spearman correlation analysis of mRNA data showed that *Adgre1* (the gene encoding F4/80) was significantly positively correlated with *Mmp2*, *Mmp14*, *Mmp9*, and *Timp1* in *WT* and *Ccr2*^*−/−*^ EAO testes indicating a connection between macrophages and MMP expression (Fig. 7H and Supplementary Fig. S6A–C).

### Integrin subunits and PDGFs expression are dysregulated in activin A-treated BMDMs and *WT* EAO testes

Integrins (receptors for ECM proteins) and PDGFs produced by immune cells contribute to ECM deposition and fibrosis development [52, 53]. Our RNAseq results suggested that activin A treatment regulated the expression of *Pdgfa* and integrin subunits *Itgb3* and *Itgav* (Fig. 5F). Therefore, we went on to investigate the expression of integrin and PDGF genes in activin A-treated BMDMs and EAO testes by qRT-PCR. In BMDMs, activin A-induced significant dose-dependent upregulation of *Itgav*, *Itgb5*, *Pdgfa*, *Pdgfb,* and *Pdgfrb* expression, whereas *Itgb3* mRNA was downregulated (Supplementary Fig. S7A and B). In *WT* EAO testes, expression of *Itgav*, *Itgb3*, *Pdgfb,* and *Pdgfrb* (Supplementary Fig. S7C, D and G, H) was also significantly increased, while upregulation of these genes was inhibited in the absence of *Ccr2*. Furthermore, expression of *Itgav* and *Pdgfrb* was positively associated with *Adgre1* levels in EAO testis (Supplementary Fig. S6D and I). These two genes showed similar changes in vitro and in vivo, indicating that activin A may regulate the production of the αv integrin subunit and PDGFRβ in macrophages, facilitating the development of testicular fibrosis. However, the levels of *Itgb5* and *Pdgfa* mRNAs were unchanged during EAO, and *Itgb5* was expressed at lower levels in *Ccr2*^*−/−*^ EAO testes, compared with *WT* EAO testes (Supplementary Fig. S7E and F).

## Discussion

In this study, we aimed to investigate the role of the activin A/CCL2-CCR2/macrophage axis in the development of testicular fibrosis. We hypothesized that activin A may be involved in the regulation of inflammatory and fibrotic responses during EAO by regulating the potential pro-fibrotic properties of newly-recruited CCR2^+^ macrophages. To examine these possibilities, we compared the progression of EAO in *WT* and *Ccr2*^*−/−*^ mice as well as in mice overexpressing follistatin to reduce activin bioactivity. Our results demonstrated that CCR2 and activin A, acting through macrophages, are crucial for the development of testicular inflammation and fibrosis in EAO. In short, testicular inflammation and fibrosis were reduced in the absence of *Ccr2*, as indicated by lower numbers of immune cells expressing ECM proteins (fibronectin, collagen I) and decreased production of collagen, MMPs, as well as inflammatory mediators (indicated by changes in *Il-10*, *Ccl2*, and *Inhba* expression). Furthermore, activin A acted as an inducer of CCR2 and fibrosis-related mediators (fibronectin, CXCR4, MMP2, and PDGF) in macrophages. Inhibition of activin A in vivo by overexpression of FST during EAO decreased testicular fibrosis and expression of collagen I by TMs.

EAO is a well-established mouse model of autoimmune-based male infertility featuring marked testicular deposition of ECM proteins (including collagens and fibronectin), increased numbers of TMs, and elevated levels of inflammatory mediators such as TNF, CCL2, and activin A. These pathological alterations combine to cause tissue destruction, inhibition of spermatogenesis, and subsequent infertility [8, 13, 21, 24]. Overall, our study revealed CCR2 and activin A to be important players in the progression of fibrosis in testicular inflammation. Furthermore, our findings underlined the pro-fibrotic role of monocyte-derived macrophages in the development of testicular fibrosis.

CCR2 is a receptor for CCL2, an inflammatory chemokine that mediates the chemotaxis of leukocytes to injury sites [54]. In this study, the absence of CCR2 during testicular inflammation not only preserved the tissue from the deposition of ECM and production of activin A, IL-10, and CCL2 but also protected the testis from damage by reducing the accumulation of immune cells. This is in accordance with previous reports demonstrating reduced inflammatory and fibrotic responses in different organs in the absence of CCR2 signaling [14, 28, 30, 55–57]. Here, we showed that in contrast to *WT* animals, *Ccr2*^*−/−*^ mice exhibited normal levels of *Il-1a*, *Il-10*, *Ccl2*, and *Inhba* mRNA in the testes following EAO induction, pointing to the essential contribution of CCR2^+^ cells to the induction of the inflammatory response.

Since the mechanisms leading to the fibrotic response and the origin of ECM proteins during the progression of testicular inflammation were unknown, we wanted to address these questions. ECM-producing cells originate from a variety of sources, including bone marrow-derived circulating cells known as fibrocytes [15, 58]. Fibrocytes are characterized by the co-expression of hematopoietic markers such as CD45, CD34, or CD11b and mesenchymal markers such as collagen or vimentin [59]. These cells mainly migrate to injury sites by chemotaxis via chemokine ligand/receptor pathways, including CCL2/CCR2 or CXCL12/CXCR4 [59, 60]. Fibrocytes can also originate from a subpopulation of monocytes via monocyte-to-fibroblast transition [61–63]. Our results indicate that during the severe stage of EAO, immune cells (particularly macrophages) act as important sources of fibronectin and collagen I production. Since the CD34 marker was not included in our analysis, we cannot definitely exclude the possibility that a proportion of the ECM-expressing CD45^+^ cells may have been fibrocytes. The significant reduction in the accumulation of CD45^+^ cells and TMs expressing fibronectin, collagen I, and CXCR4 observed in EAO *Ccr2*^*−/−*^ testis indicates the importance of CCR2 and CXCR4 signaling in testicular ECM expression. In addition, the very high levels of *Cxcl12* and *Cxcr4* mRNA detected in *WT* EAO testis were significantly reduced in the absence of *Ccr2*, underlining the importance of newly-recruited CCR2^+^ cells as a source of these molecules during the testicular fibrotic response. Similarly, the expression of fibronectin in TMs under pathological conditions in the human testis (impaired spermatogenesis and collagen accumulation) points to the possible involvement of macrophages in fibrotic remodeling. These data are in agreement with earlier reports demonstrating CCR2-dependent inhibition of BMDM and fibrocyte infiltration in kidney injury, colon fibrosis, and colitis [28, 30, 64–66]. Moreover, CXCR4^+^ macrophages have also been shown to act as a pro-fibrotic cell subpopulation in the kidney and lung [67–69]. Future studies are required to establish whether CCR2^+^CXCR4^+^ TMs are the key players involved in the EAO fibrotic response. However, currently, we cannot exclude the possibility that the production of *Cxcl12* by CCR2^+^ cells may account for the reduced infiltration of CXCR4^+^ cells in *Ccr2*^*−/−*^ mouse testes.

Our results also showed that expression of *Mmp2*, *Mmp14*, *Mmp9,* and *Timp1* was increased in *WT* EAO testes, indicating activation of the ECM machinery. The gelatinases MMP2 and MMP9 mainly cleave collagen IV, an important component of the basement membrane, making it easier for leukocytes to transmigrate [70, 71]. Activation of MMP2 requires the involvement of MMP14 [72]. Aggregated ECM proteins require degradation by MMPs to prevent fibrosis; on the other hand, TIMP1 inhibits the degradative function of MMP proteins, thereby exacerbating the fibrotic process. However, elevated levels of MMPs could also contribute to immune cell migration or activate PTCs /fibroblasts to produce more ECM proteins, thereby promoting tissue fibrosis [73, 74]. Thus, the process of testicular fibrosis appears to depend on the dynamic balance between MMP and TIMP activities. In this study, loss of *Ccr2* abrogated the elevated expression of *Mmp2* and *Mmp14* occurring in EAO testes. Interestingly, the expression of these genes positively correlated with levels of *Adgre1* mRNA, indicating that macrophages are involved in MMP and TIMP1 production and thereby in the promotion of testicular fibrosis at the inflammation stage.

The changes in integrins and PDGF gene expression observed in EAO may also contribute to the fibrotic phenotype, as these groups of molecules are known as important promoters of fibrosis [52, 53, 75, 76]. The increased levels of *Itgav*/*Itgb3* (encoding integrin αvβ3) and *Pdgfb/Pdgfrb* mRNAs detected during EAO (dependent on *Ccr2* expression) were likely due to the infiltration of macrophages, as *Itgav* and *Pdgfrb* correlated positively with *Adgre1* expression. Furthermore, PDGF acts as a strong chemoattractant for fibrocytes in pulmonary fibrosis, while pharmacological blockade of the PDGF/PDGFR axis is considered a promising treatment option [77].

**Fig. 8:**
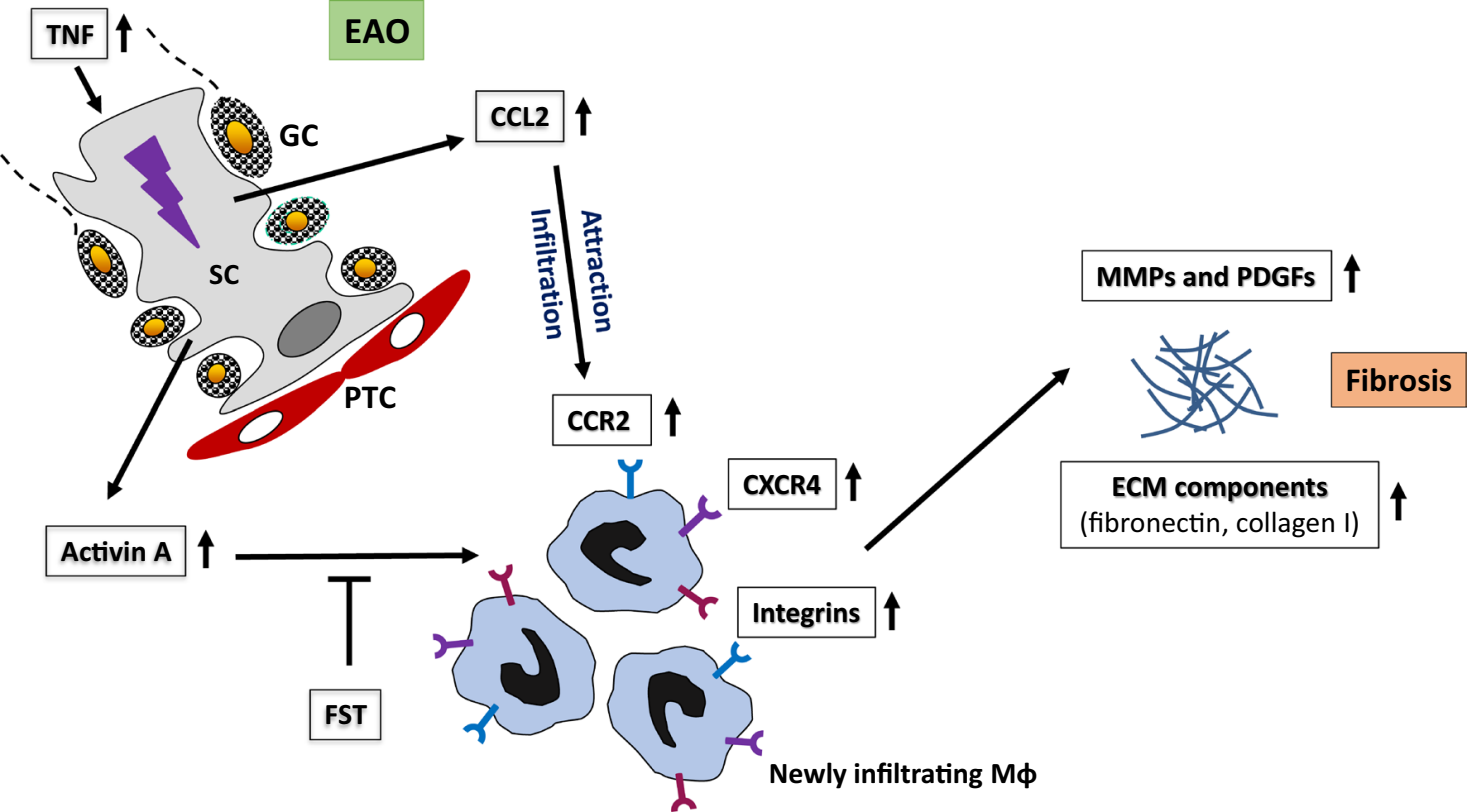
Model of the role of activin A, CCL2/CCR2, and macrophages in fibrotic remodeling during EAO. Increased expression of TNF during testicular inflammation induces SCs to release higher levels of CCL2 and activin A. In response to elevated levels of CCL2, macrophages expressing CCR2 are attracted to the site of injury and infiltrate the inflamed testes. Activin A stimulates CCR2 expression on macrophages and drives them to a pro-fibrotic phenotype by inducing the expression of CXCR4, collagen I, fibronectin, MMPs, integrins and PDGFs. Together, these processes facilitate the development of fibrosis in EAO testes. In EAO mice, inflammatory response and fibrotic remodeling can be inhibited by the knockout of *Ccr2*, blocking macrophage infiltration, or by the use of FST, an activin A antagonist. (*GC* dying germ cells, *SC* Sertoli cells, *PTC* peritubular cells, *Mφ* macrophages)

Taken together, these in vivo data indicate that CCR2 contributes to the development of testicular fibrosis during inflammation by mediating leukocyte infiltration, cytokine release, and ECM protein production by immune cells, particularly TMs. In addition, our results suggest that CCR2 + TMs may produce multiple factors, including the CXCL12/CXCR4 axis, MMPs, αv integrin, PDGFB, and PDGFRβ, which potentially act as downstream signaling molecules to regulate immune processes during testicular inflammation (summarized in Fig. 8).

In addition to CCR2, activin A is another important fibrotic regulator [6, 23]. In the current study, activin A stimulated CCR2 expression (at the mRNA and protein level) in macrophages and reduced *Ccl2* levels. Similarly opposing effects of activin A on *Ccr2* and *Ccl2* expression have been demonstrated in human monocyte-derived macrophages [78]. Our findings indicated that activin A can increase the responsiveness of inflammatory BMDMs to CCL2 by enhancing the expression of *CCR2*. Furthermore, according to our results, activin A also induced the expression of CXCR4 in macrophages. As CXCR4 is highly expressed by macrophages during testicular inflammation, reduced infiltration by monocytes/macrophages in *Ccr2*^*−/−*^ mice during EAO may explain why the level of testicular *Cxcr4* mRNA did not increase in CCR2-depleted mice following induction of EAO (see Fig. 8).

Previously, we showed that activin A-induced the expression of fibrosis-specific genes in mouse PTCs and NIH 3T3 fibroblasts. Furthermore, expression of the activin dimer subunit was increased, not only in inflamed mouse testes but also in human testes with impaired spermatogenesis and focal leukocytic infiltrates [13, 24]. Notably, the severity of the induced inflammation and damage was directly proportional to the level of activin A [13, 24]. These findings, together with the effects of *Ccr2* deficiency during EAO (including reduced infiltration of macrophages, lower levels of ECM deposition and *Inhba*) implicate activin A as a key regulator of testicular inflammation and fibrosis, acting through TMs. In vivo inhibition of activin A by overexpression of follistatin clearly demonstrated the role of activin A in the development of testicular inflammation; for example, suppression of the accumulation of TM-derived collagen I by follistatin indicated that activin A affected the progression of testicular fibrosis by regulating pro-fibrotic properties of macrophages. Moreover, the expression of *Inhba* correlated positively with testicular *Mmp* and *Timp1* expression during EAO. Therefore, elevated levels of MMPs and TIMP1 in EAO testes were associated with both macrophage infiltration and activin A expression.

Activin A treatment influenced the transcriptome of BMDMs in vitro and increased the expression of *Fn1*, *Ccr2*, *Cxcr4, Mmp2*, *Mmp14*, *Itgav*, *Pdgfa*, *Pdgfb*, and *Pdgfrb* genes as well as the levels of fibronectin, CCR2, CXCR4, and MMP2 proteins. Moreover, activin A enhanced the migratory ability of macrophages in response to CCL2 stimulation. Interestingly, in addition to increased expression of fibrosis-related genes, activin A-induced transformation to spindle-shaped morphology and diminished F4/80 fluorescence in BMDMs, indicating possible conversion to a fibroblastic phenotype in vitro. Similarly, activin A reportedly reprogrammed pre-tumorigenic macrophages to a tumor-associated phenotype, promoted conversion of CD4^+^CD25^−^ naïve T cells into regulatory T cells, and induced a pro-fibrotic transcriptome in fibroblasts, thereby transforming them into myofibroblasts [79–81]. Furthermore, macrophages from the synovium of active rheumatoid arthritis patients, which exhibited pro-inflammatory, macrophage-polarizing properties, were inhibited by the addition of an anti-activin A neutralizing antibody [82].

Combining these results, we postulate that activin A promotes the progression of testicular fibrosis by inducing the production of fibrosis-related mediators in macrophages. Furthermore, although activin A treatment reduced the expression of *Mmp9*, *Timp1,* and *Itgb3* in BMDMs, other factors, such as TNF, may increase the levels of MMP9 and TIMP1 in macrophages or other testicular cells, thereby promoting their expression in EAO testes [83]. In addition, cells such as fibroblasts can produce large amounts of β3 integrin in response to inflammatory stimuli, which could underlie the high levels of *Itgb3* mRNA observed in EAO testes [84].

Critically, it should be noted that utilizing BMDMs matured in the presence of M-CSF as a surrogate for TMs may not exactly replicate all functional aspects of the TMs. However, M-CSF is a critical regulator of the maturation and tissue-specific functions of TMs [85]. As TMs make up a very small proportion of the normal mouse testis cell population, in vitro experimentation on isolated TMs would require an unreasonably large number of mice. In accordance with 3R principles, TMs were replaced by BMDMs, drastically reducing the number of animals required.

Taken together, our in vivo and in vitro results illustrate that activin A promotes the differentiation of macrophages to a pro-fibrotic phenotype through the induction of a variety of inflammatory mediators and receptor molecules (summarized in Fig. 8). Activin A-stimulated BMDMs are potential fibroblast precursors, which likely facilitate tissue fibrosis. In addition, by inducing CCR2 expression, activin A potentially affects the recruitment of monocytes/macrophages to injury sites. Although our data indicate that CCR2, activin A, and macrophages are responsible for the testicular fibrotic response, the precise immune mechanisms that regulate this process require further investigation. Future studies are needed to address the direct influence of activin A using targeted depletion of activin A receptors (in monocytes/macrophages) to analyze their role in inflammatory responses and fibrotic remodeling.

In conclusion, our data indicate that CCR2 and activin A regulate the development of fibrosis during testicular inflammation and underline the crucial pro-fibrotic function of macrophages in inflammation-associated fibrotic remodeling in EAO. Future therapeutic targeting of CCR2 and/or activin A may offer a possible strategy to address testicular fibrosis.

## Supplementary Information

Below is the link to the electronic supplementary material.

Supplementary file1 (PDF 1997 KB)

## Data Availability

The data presented in this study are available in the article and supplementary material. Transcriptome data are available online at the GEO repository (https://www.ncbi.nlm.nih.gov/geo/query/acc.cgi?acc=GSE210004).

## Abbreviations

αSMA: *α*-Smooth muscle actin
BMDM: Bone marrow-derived macrophage
CCL2: C–C motif chemokine ligand 2
CCR2: C–C motif chemokine receptor type 2
DEGs: Differentially expressed genes
EAO: Experimental autoimmune orchitis
ECM: Extracellular matrix
FST: Follistatin
IL: Interleukin
MMP: Matrix metalloproteinase
PDGF: Platelet-derived growth factor
PTC: Peritubular cell
SC: Sertoli cell
SCCM: Sertoli cell-conditioned medium
TGF-β: Transforming growth factor-β
TIMP: Tissue inhibitor of metalloproteinase
TMs: Testicular macrophages
TNF: Tumor necrosis factor
WT: Wild type
