# Supplementary material for: Activin A and CCR2 regulate macrophage function in testicular fibrosis caused by experimental autoimmune orchitis
Source: Cell Mol Life Sci. 2022 Nov 24;79(12):602. doi: 10.1007/s00018-022-04632-4 (PMC9700630; doi:10.1007/s00018-022-04632-4)
Supplement: Supplementary file 1 — Supplementary file1 (PDF 1997 KB) [file 18_2022_4632_MOESM1_ESM.pdf]

Supplementary Files

Figure S1

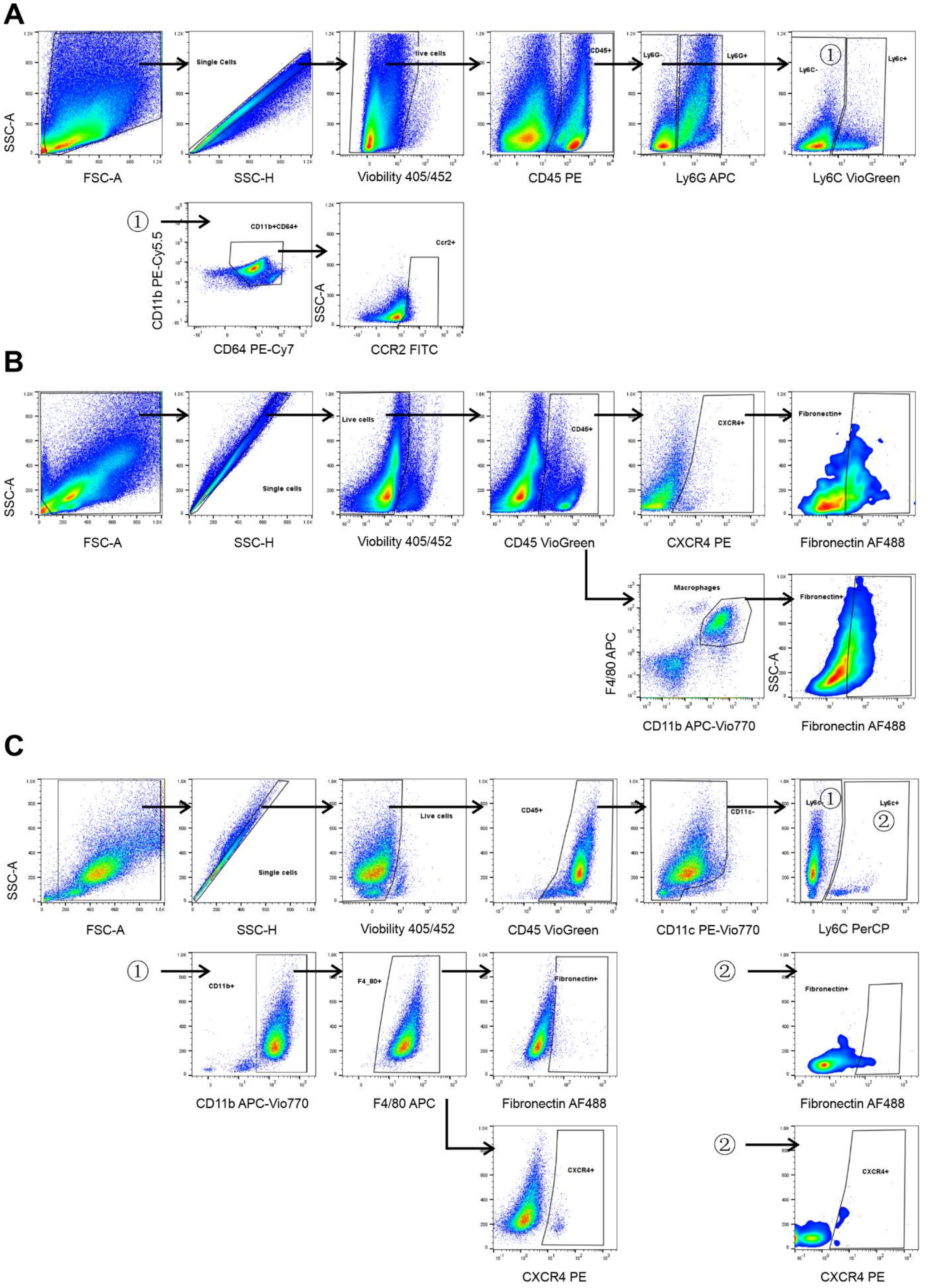

Supplementary Figure S1. Flow cytometry gating strategy to analyze testicular macrophages and BMDMs.

Representative flow cytometry plots show the gating scheme used for the analysis of immune cell subpopulations **(A)** and ECM-expressing immune cells **(B)** in untreated, adjuvant control, and EAO testes from *WT* and *Ccr2*$^{-/-}$ mice. The gating strategy for fibronectin^{+} and CXCR4^{+} cells in cultured BMDMs after activin A or FST288 treatments is also shown **(C)** (SSC: side scatter; FSC: forward scatter).

Figure S2

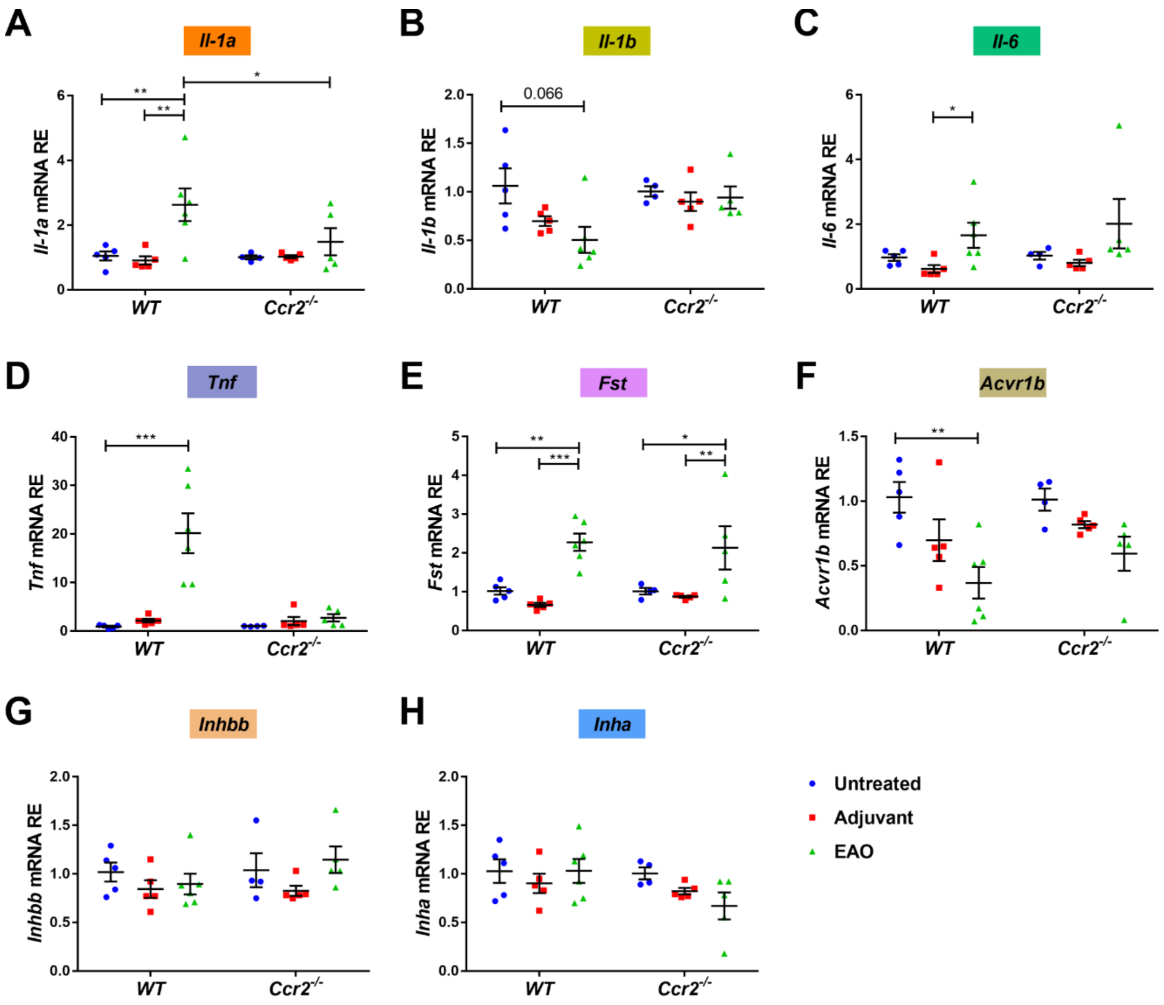

Supplementary Figure S2. Expression of cytokines, follistatin, activin receptor and activin subunits in untreated, adjuvant control, and EAO testes from *WT* and *Ccr2*$^{-/-}$ mice.

Relative expression (RE) of *Il-1a* **(A)**, *Il-1b* **(B)**, *Il-6* **(C)**, *Tnf* **(D)**, *Fst* **(E),** and *Acvr1b* **(F),** *Inhbb* **(G)**, and *Inha* **(H)**, was determined by qRT-PCR in untreated, adjuvant control, and EAO testes from *WT* and *Ccr2*$^{-/-}$ mice. Data were normalized to *18S rRNA* and *Hprt* (n = 4–6). Values are mean ± SEM; the Kruskal-Wallis test followed by Dunn's multiple comparison test or two-way ANOVA followed by Bonferroni's multiple comparison test was employed for statistical analysis. **P* < 0.05, ***P* < 0.01, ****P* < 0.001.

Figure S3

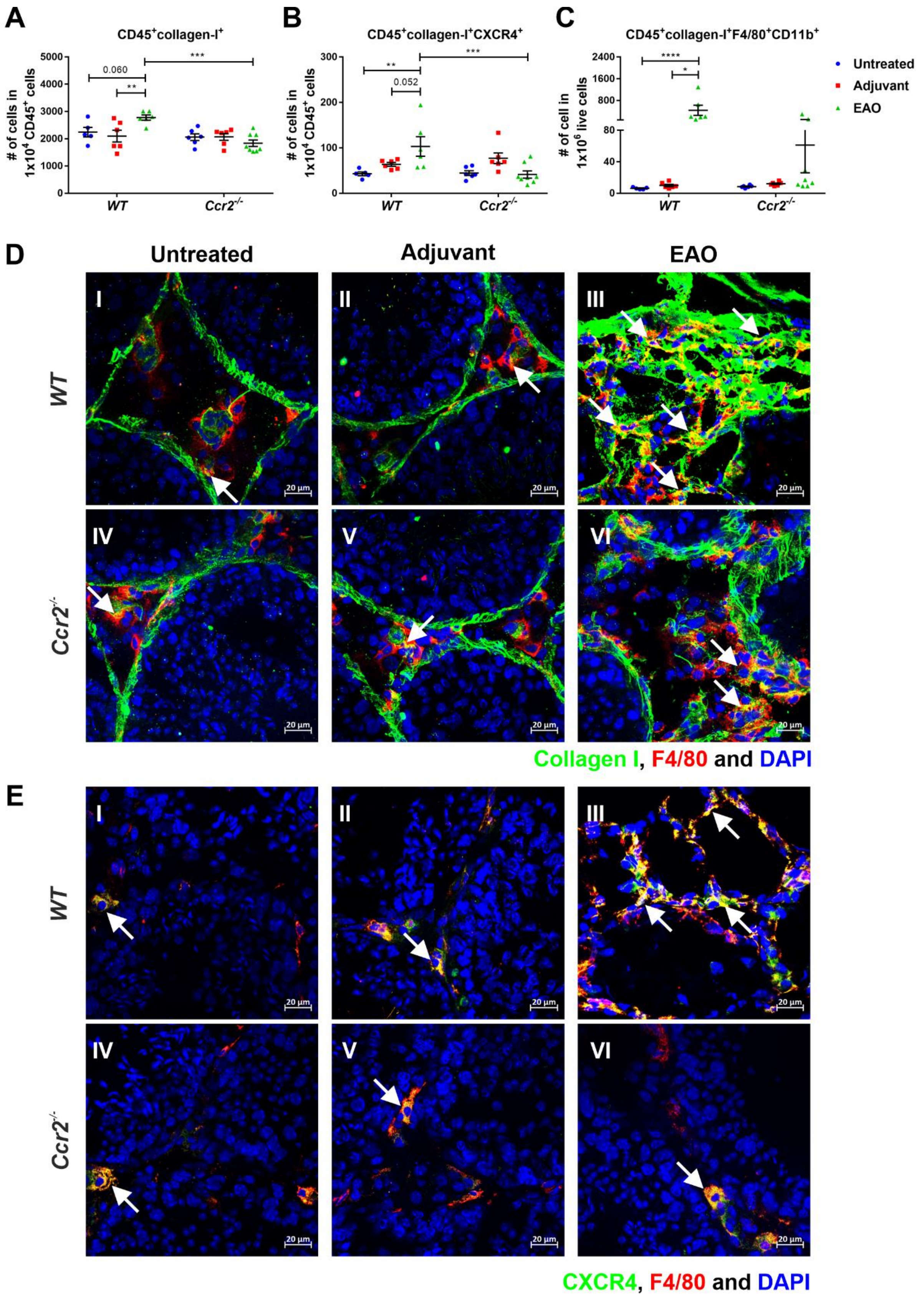

Supplementary Figure S3. Quantification of collagen I$^+$ and CXCR4$^+$ F4/80 macrophages in untreated, adjuvant control, and EAO testes from *WT* and *Ccr2$^{-/-}$* mice.

After gating out doublets and nonviable cells, the numbers of CD45$^+$collagen I$^+$ cells **(A)** and CD45$^+$collagen I$^+$CXCR4$^+$ cells **(B)** within the CD45$^+$ leukocyte population and the number of CD45$^+$collagen I$^+$F4/80$^+$CD11b$^+$ cells **(C)** within the live cell population were analyzed by flow cytometry in untreated, adjuvant control, and EAO testicular single-cell suspensions from *WT* and *Ccr2$^{-/-}$* mice (n = 5–8). Representative photomicrographs of collagen I (green), F4/80 (red), and DAPI (blue) **(D)**, and CXCR4 (green), F4/80 (red), and DAPI (blue) **(E)** triple immunofluorescence staining in frozen sections from untreated **(I, IV)**, adjuvant control **(II, V),** and EAO **(III, VI)** testes in *WT* **(I–III)** and *Ccr2$^{-/-}$* **(IV–VI)** mice; arrows indicate triple-positive cells. Values are mean ± SEM; the Kruskal-Wallis test followed by Dunn's multiple comparison test or two-way ANOVA followed by Bonferroni's multiple comparison test was employed for statistical analysis. **P* < 0.05, ***P* < 0.01, ****P* < 0.001, *****P* < 0.0001.

Figure S4

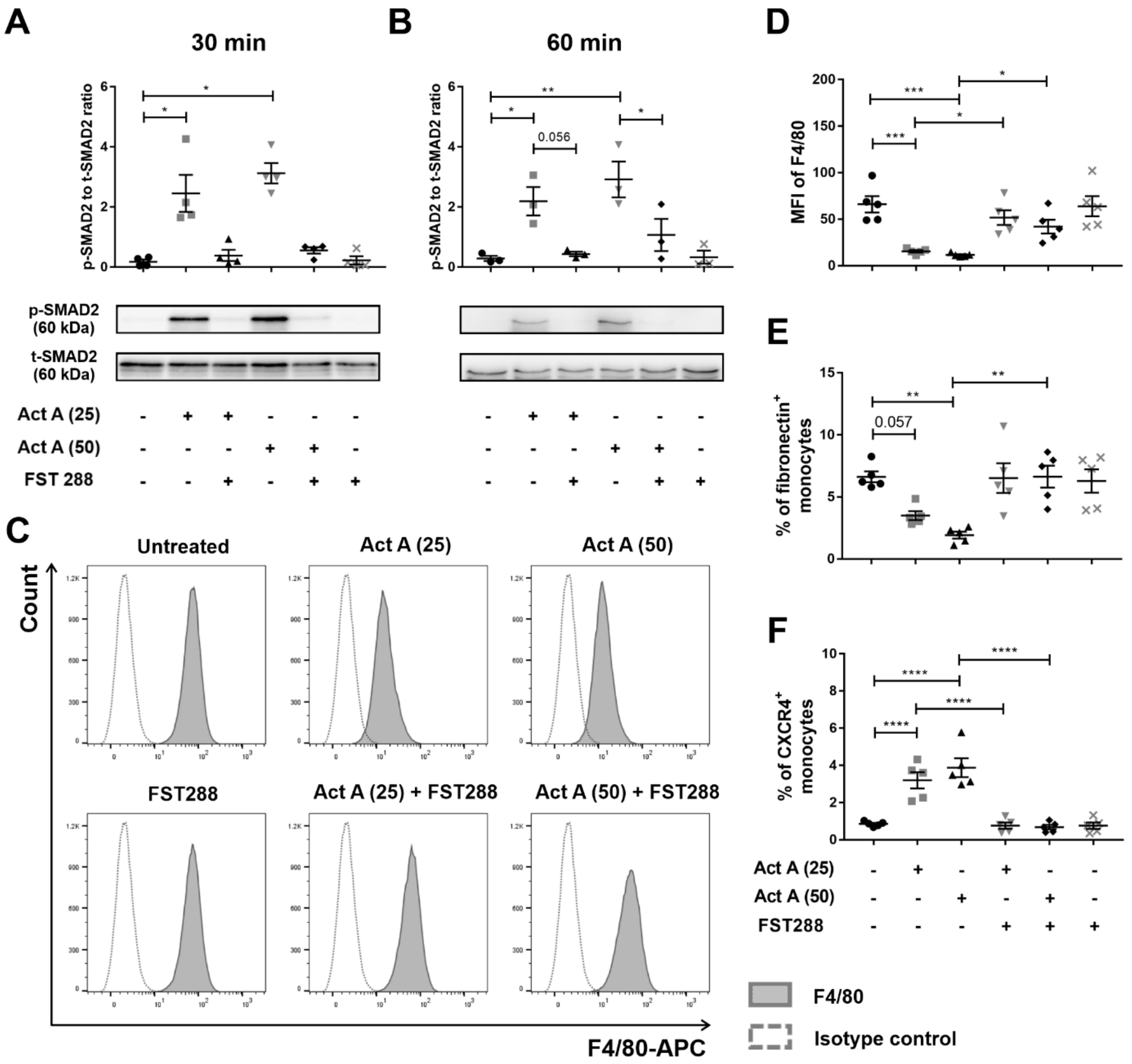

Supplementary Figure S4. Phosphorylation of SMAD2, levels of F4/80 mean fluorescence intensity, and percentages of fibronectin$^+$ and CXCR4$^+$ monocytes following activin A stimulation in BMDMs.

BMDMs were treated with 25 ng/50 ng/ml activin A, 250 ng/ml FST288, or a combination of both, and phosphorylation of SMAD2 was analyzed after 30 min **(A;** n = 4**)** or 60 min **(B;** n = 3**)** by Western blotting. Total (t)-SMAD2 was used as a loading control. After gating CD45$^+$CD11c$^-$Ly6C$^-$F4/80$^+$CD11b$^+$ macrophages or CD45$^+$CD11c$^-$Ly6C$^+$ monocytes, the mean fluorescence intensity (MFI) of F4/80 cells **(C, D;** n = 5**)** and the percentages of CD45$^+$CD11c$^-$Ly6C$^+$fibronectin$^+$ **(E;** n = 5**)** and CD45$^+$CD11c$^-$Ly6C$^+$CXCR4$^+$ **(F;** n = 5**)** cells in single-cell BMDM suspensions after treatment with 25 ng/50 ng/ml activin A, 250 ng/ml FST288, or a combination of both were analyzed by flow cytometry. Values are mean ± SEM; the Kruskal-Wallis test followed by Dunn's multiple comparison test or one-way ANOVA followed by Bonferroni's multiple comparison test was employed for statistical analysis. **P* < 0.05, ***P* < 0.01, ****P* < 0.001, *****P* < 0.0001.

Figure S5

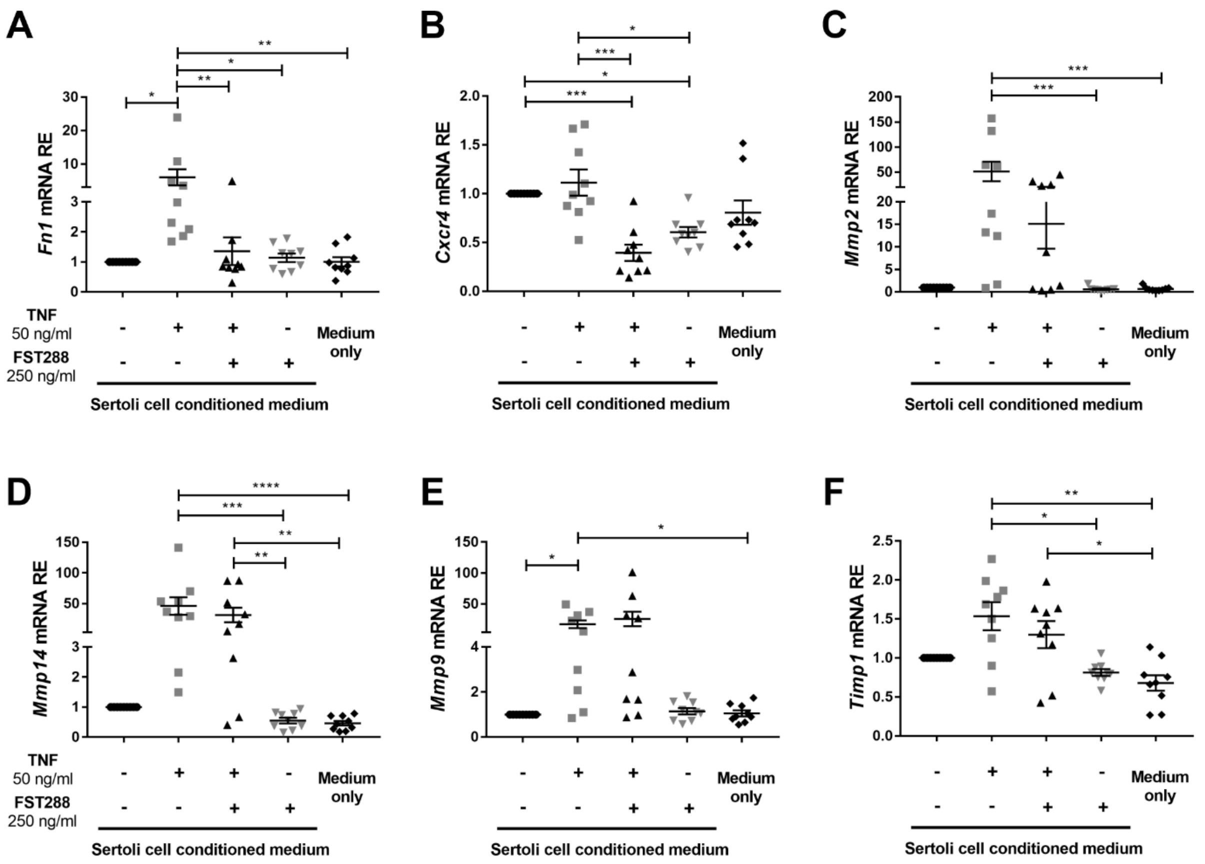

Supplementary Figure S5. The expression of fibrosis-related genes in BMDMs after incubation with SCCM.

Relative expression (RE) of *Fn1* **(A)**, *Cxcr4* **(B)**, *Mmp2* **(C)**, *Mmp14* **(D)**, *Mmp9* **(E),** and *Timp1* **(F)** in BMDMs 3 days after treatment with conditioned medium from SCs cultivated alone or in the presence of 50 ng/ml TNF, 250 ng/ml FST288, or a combination of both was determined by qRT-PCR analysis and normalization to *Hprt* (n = 9). Values are mean ± SEM; the Kruskal-Wallis test followed by Dunn's multiple comparison test was employed for statistical analysis. *$P < 0.05$, **$P < 0.01$, ***$P < 0.001$, ****$P < 0.0001$.

Figure S6

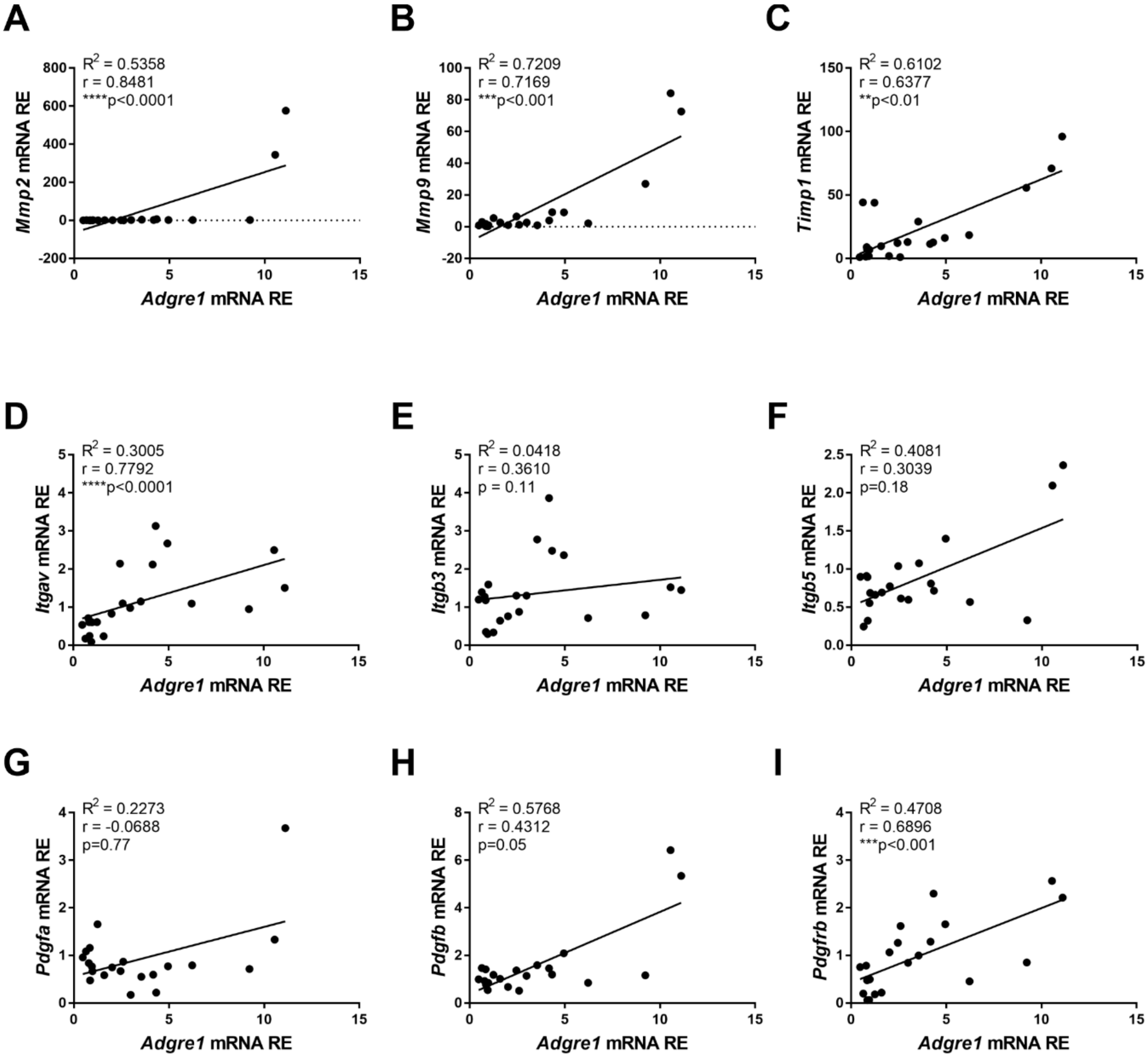

Supplementary Figure S6. Correlation analyses of *Adgre1* with *Mmp2, Mmp9, Timp1, Itgav,* and *Pdgfrb* mRNAs in *WT* and *Ccr2*$^{-/-}$ EAO testes.

Relative expression (RE) of *Adgre1* mRNA was determined by qRT-PCR analysis and normalization to *18S rRNA* and *Hprt* (n = 7–13). The correlations between *Adgre1* and *Mmp2* **(A)**, *Mmp9* **(B)**, *Timp1* **(C)**, *Itgav* **(D)**, *Itgb3* **(E)**, *Itgb5* **(F)**, *Pdgfa* **(G)**, *Pdgfb* **(H)** and *Pdgfrb* **(I)** mRNAs in *WT* and *Ccr2*$^{-/-}$ EAO testes were analyzed. Correlation significance was determined using Spearman's coefficient. **P* < 0.05, ***P* < 0.01, ****P* < 0.001, *****P* < 0.0001.

Figure S7

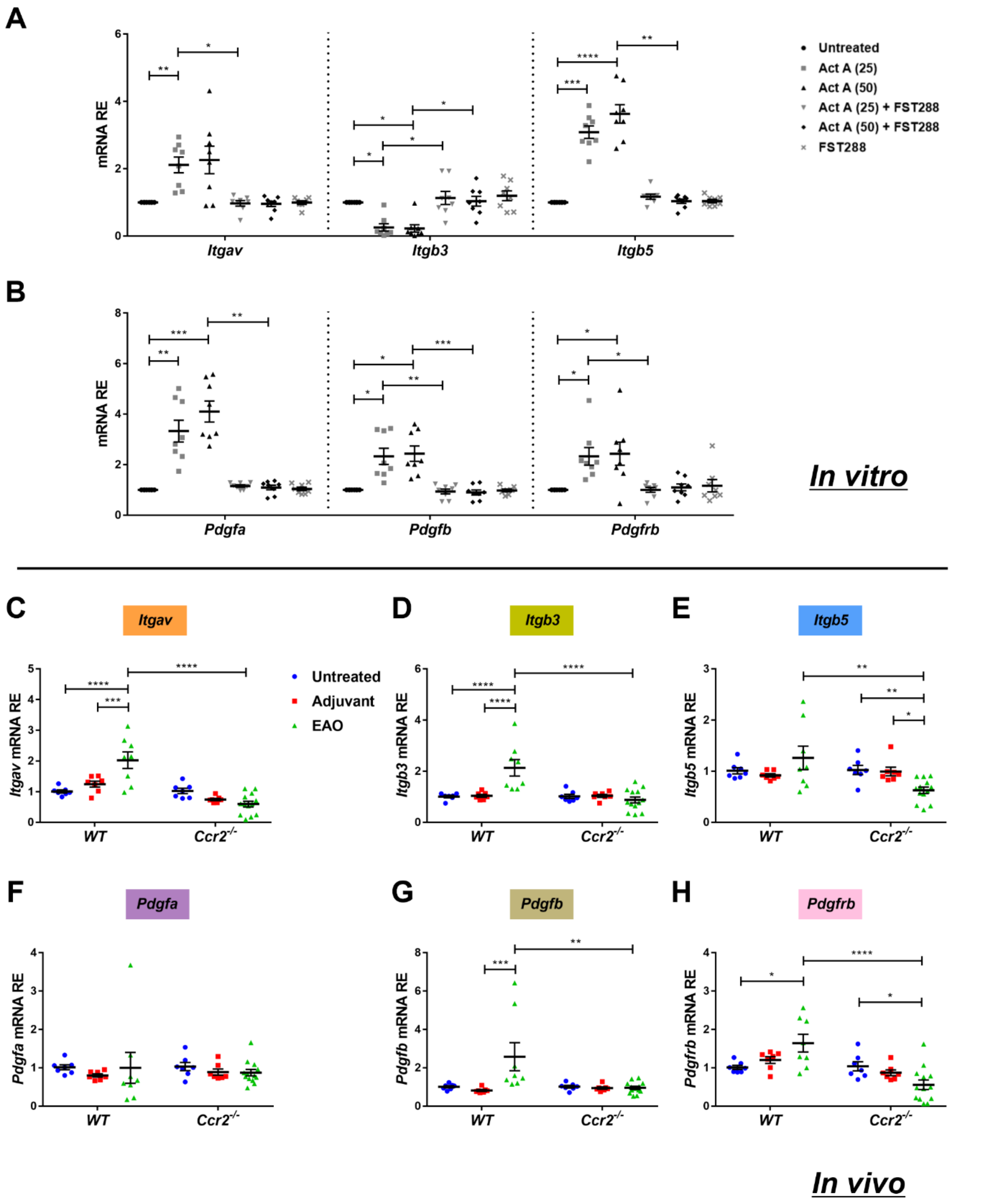

Supplementary Figure S7. Expression of integrin subunits and PDGFs is altered in activin A-treated BMDMs *in vitro* and in EAO testes *in vivo*.

Relative expression (RE) of *Itgav*, *Itgb3,* and *Itgb5* **(A)** and *Pdgfa*, *Pdgfb,* and *Pdgfrb* **(B)** in BMDMs 6 days after treatment with 25 ng/50 ng/ml activin A, 250 ng/ml FST288, or a combination of both was determined by qRT-PCR analysis and normalization to *Hprt* mRNA (n = 8). Similarly, RE of *Itgav* **(C)**, *Itgb3* **(D)**, *Itgb5* **(E)**, *Pdgfa* **(F)**, *Pdgfb* **(G),** and *Pdgfrb* **(H)** in untreated, adjuvant control, and EAO testes from *WT* and *Ccr2*$^{-/-}$ mice was determined by

qRT-PCR analysis and normalization to *18S rRNA* and *Hprt* (n = 7–13). Values are mean ± SEM; the Kruskal-Wallis test followed by Dunn's multiple comparison test or one-way/two-way ANOVA followed by Bonferroni's multiple comparison test was employed for statistical analysis. *$P < 0.05$, **$P < 0.01$, ***$P < 0.001$, ****$P < 0.0001$.
